# Supplementary material for: Integrating Multiple Omics Identifies Phaeoacremonium rubrigenum Acting as Aquilaria sinensis Marker Fungus to Promote Agarwood Sesquiterpene Accumulation by Inducing Plant Host Phosphorylation
Source: Microbiol Spectr. 2022 Jun 28;10(4):e02722-21. doi: 10.1128/spectrum.02722-21 (PMC9431625; doi:10.1128/spectrum.02722-21)
Supplement: Supplemental file 1 — Supplemental material. Download spectrum.02722-21-s0001.pdf, PDF file, 2.6 MB [file spectrum.02722-21-s0001.pdf]

SUPPLEMENTAL FILE

Integrating multiple omics identifies *Phaeoacremonium rubrigenum* acting as *Aquilaria sinensis* marker fungus to promote agarwood sesquiterpene accumulation by inducing plant host phosphorylation

Liu *et al.*

Supplementary figures and tables

Supplementary Figure S1	Light and fluorescent microscopic features of fresh agarwood samples.
Supplementary Figure S2	Fungal species diversity analysis.
Supplementary Figure S3	Stacking histogram of fungal distribution associated with the five layers of agarwood.
Supplementary Figure S4	Correlation network of all detected fungi with sesquiterpenes.
Supplementary Figure S5	Phylogenetic analysis of our isolated fungal strain and *Phaeoacremonium* sp. using ITS sequences.
Supplementary Figure S6	Gas chromatography-mass spectroscopy profiles of *P. rubrigenum* hyphae and fermentation media.
Supplementary Figure S7	Heatmap of protein phosphorylation levels of TFs in *A. sinensis*.
Supplementary Table S1	Sesquiterpene and chromone compounds were identified in the different layers of wounded trees using GC-MS.
Supplementary Table S2	Data pre-processing statistics and quality control of high-throughput fungal sequencing diversity in different layers of agarwood.
Supplementary Table S3	Tukey analysis of significant difference of diversity index among different groups.
Supplementary Table S4	Sesquiterpene and chromone compounds identified in *P. rubrigenum*-treated *A. sinensis* stems using GC-MS.
Supplementary Table S5	Sesquiterpene compounds identified in *P. rubrigenum*-treated *A. sinensis* calli using GC-MS.
Supplementary Table S6	Relative TMT protein expression quantities of the proteins involved in sesquiterpene biosynthesis.
Supplementary Table S7	Relative TMT protein expression quantities of the proteins involved in transcription factor.
Supplementary Table S8	Relative protein phosphorylation quantities of the proteins involved in transcription factor.
Supplementary Table S9	The binding sites of TFs in the promoters of genes involved in sesquipterpene biosynthesis in *A. sinensis*.

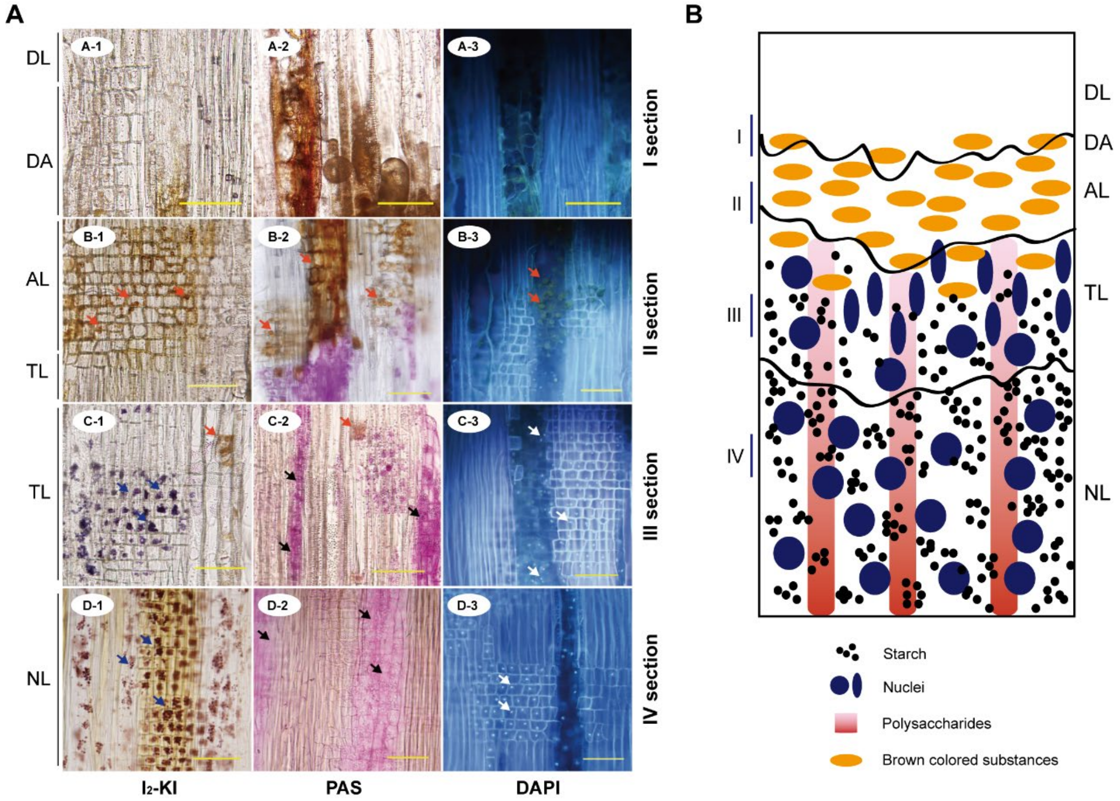

Fig. S1. Light and fluorescent microscopic features of fresh agarwood samples. (A) Micrographs of different sections, showing distribution pattern of starch grains, polysaccharides, brown resin-like materials and live cells. AL, the agarwood layer; TL, the agarwood-normal transition layer; NL, the normal layer. I section, partial DL and partial DA; II section, partial AL and partial TL; III section, TL; IV section, NL. A-1 shows the brown resin-like materials and starch grains in I section by using I_2-KI staining; A-2 shows the brown resin-like materials and polysaccharides in I section by using Periodic Acid Schiff (PAS) staining; A-3 shows the brown resin-like materials and nuclei in I section by using DAPI staining; B-1 shows the brown resin-like materials and starch grains in II section by using I_2-KI staining; B-2 shows the brown resin-like materials and polysaccharides in II section by using Periodic Acid Schiff (PAS) staining; B-3 shows the brown resin-like materials and nuclei in II section by using DAPI staining; C-1 shows the brown resin-like materials and starch grains in III section by using I_2-KI staining; C-2 shows the brown resin-like materials and polysaccharides in III section by using PAS staining; C-3 shows the brown resin-like materials and nuclei in III section by using DAPI staining; D-1 shows the brown resin-

like materials and starch grains in IV section by using I_2-KI staining; D-2 shows the brown resin-like materials and polysaccharides in IV section by using PAS staining; D-3 shows the brown resin-like materials and nuclei in IV section by using DAPI staining. White arrowheads indicate nuclei; red arrowheads indicate brown resin-like materials; blue arrowheads indicate starch grains; black arrowheads indicate polysaccharides. Scale bars = 100 μm. **(B)** A diagram on distribution patterns of storage starch, polysaccharides, brown resin-like materials and nuclei in different layers of wounded wood. DL, the dacay layer; DA, the decay-agarwood transition layer; AL, the agarwood layer; TL, the agarwood-normal transition layer; NL, the normal layer. Label I shows the site of micrographs of A1~A3 in Fig. S1A; Label II shows the site of micrographs of B1~B3 in Fig. S1A; Label III shows the site of micrographs of C1~C3 in Fig. S1A; Label IV shows the site of micrographs of D1~D3 in Fig. S1A.

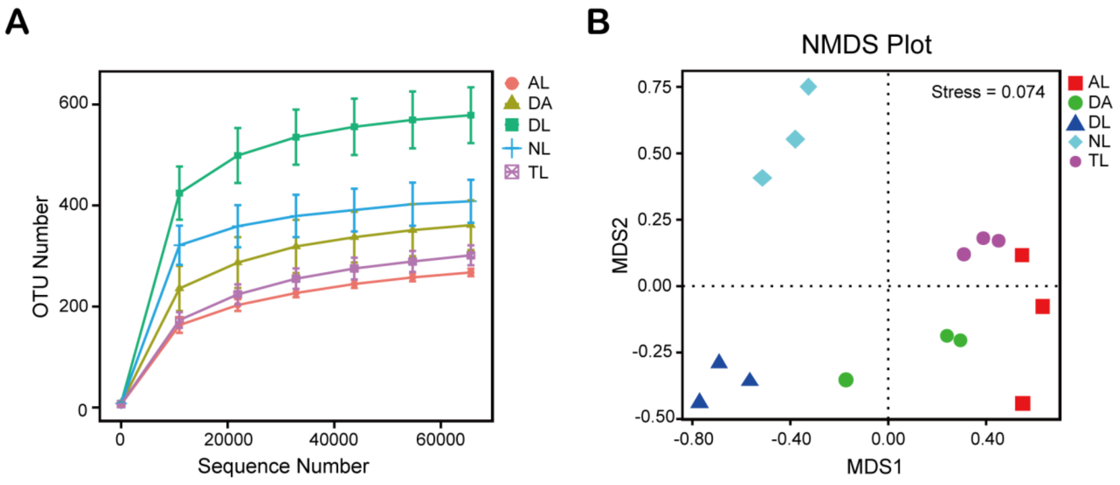

Fig. S2. Fungal species diversity analysis. (A) A rarefaction curve of OTUs shows that the amount of sequencing data is close to saturation, and the curve trend shows the species diversity. **(B)** An NMDS plot revealing fungal community diversity by nonlinear structure.

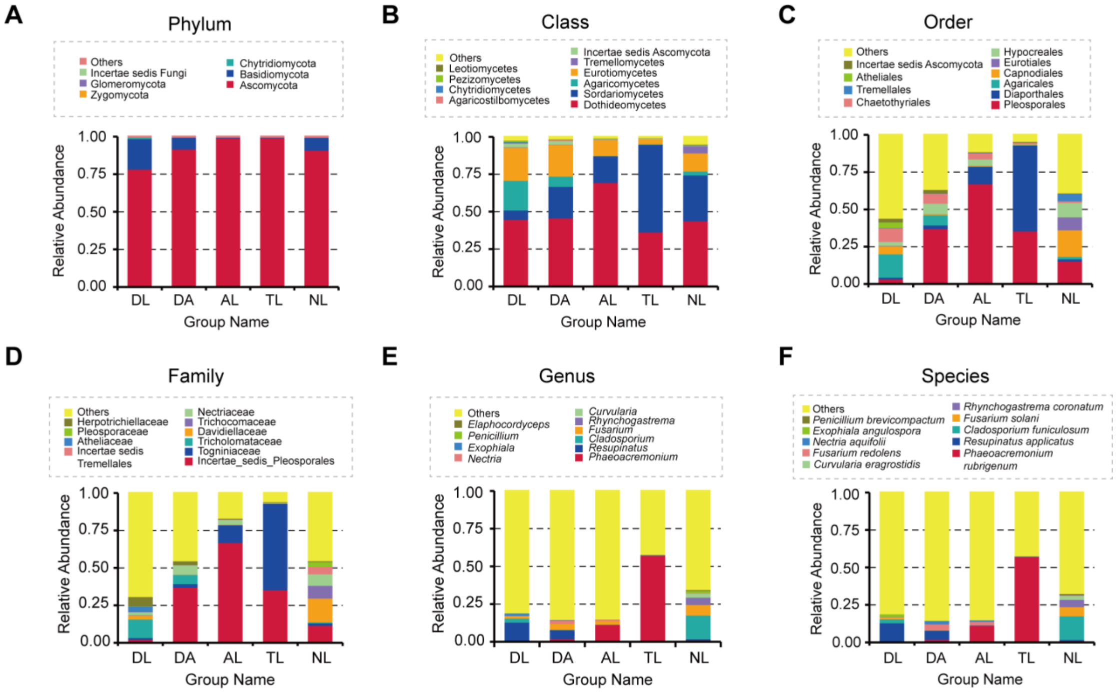

Fig. S3. Stacking histogram of fungal distribution associated with the five layers of agarwood. The figures showed the top 10 fungal taxa in different levels, including phylum **(A)**, class **(B)**, order **(C)**, family **(D)**, genus **(E)**, and species **(F)**.

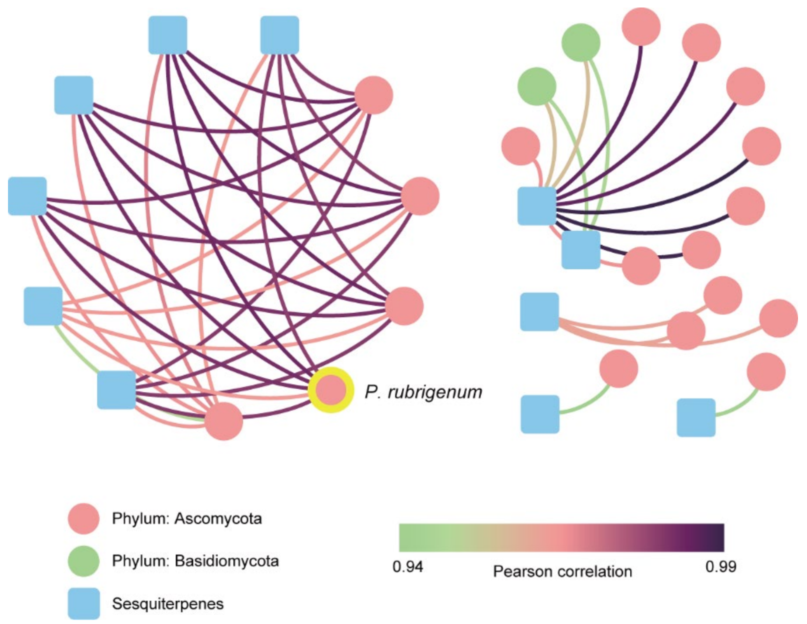

Fig. S4. Correlation network of all detected fungi with sesquiterpenes. Line color represents the computed pearson correlation values. Only relations with the squared correlation value larger than 0.6 and multiple test corrected p-value less than 0.05 were kept in the network. Circles represent phylums of fungi and squares represent sesquiterpenes. *P. rubrigenum* is highlighted.

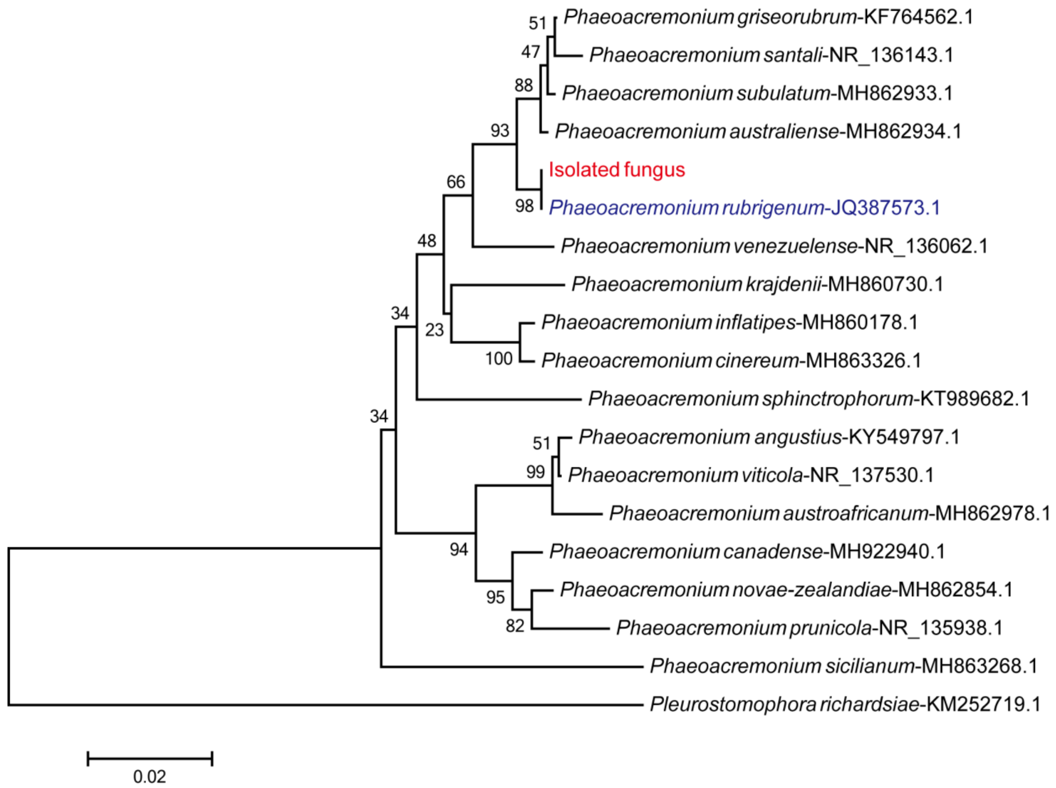

Fig. S5. Phylogenetic analysis of our isolated fungal strain and *Phaeoacremonium* sp. using ITS sequences.

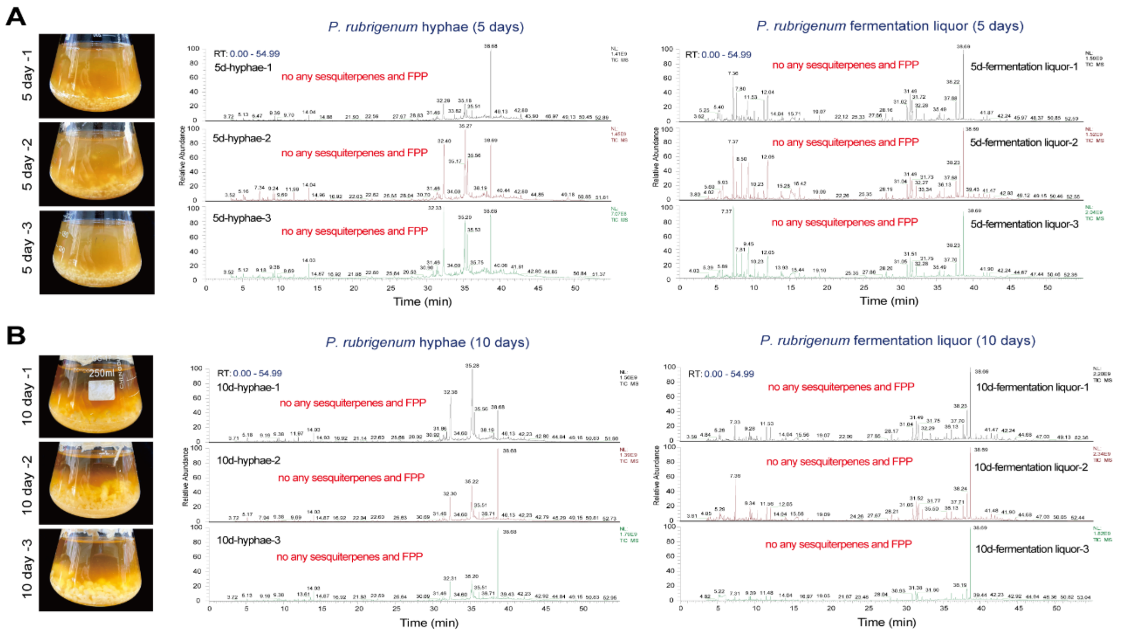

Fig. S6. Gas chromatography-mass spectroscopy profiles of *P. rubrigenum* hyphae and fermentation media. (A) Total ion chromatogram of the volatile compounds of 5-day cultured *P. rubrigenum* hyphae and fermentation media. n=3. There is no any sesquiterpenes and FPP detected. **(B)** Total ion chromatogram of the volatile compounds of 10-day cultured *P. rubrigenum* hyphae and fermentation media. n=3. There is no any sesquiterpenes and FPP detected.

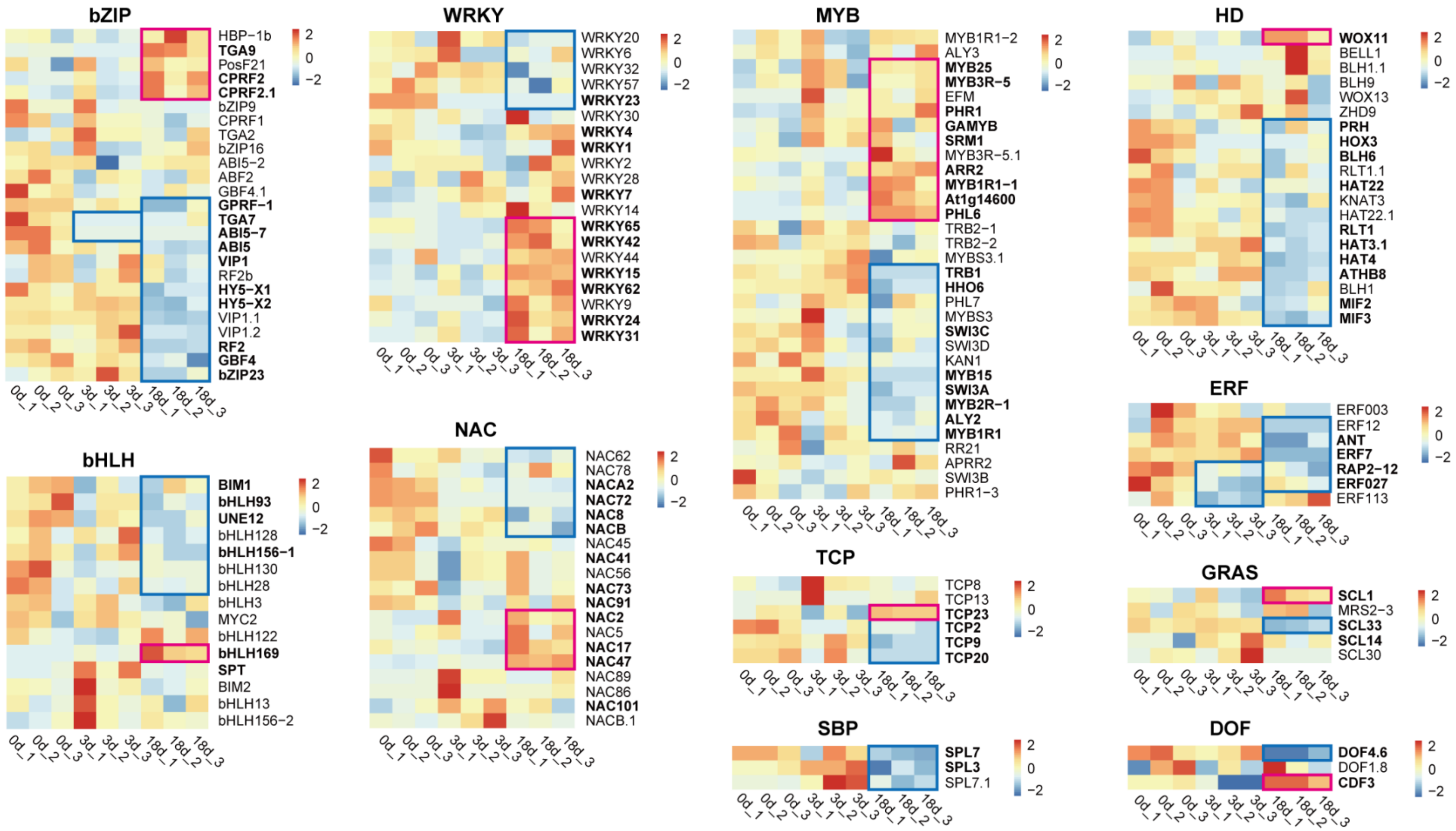

Fig. S7. Heatmap of protein phosphorylation levels of TFs. Red frame, upregulated TFs' phosphorylation; blue frame, downregulated TF's phosphorylation.

Table S1. Sesquiterpene and chromone compounds identified in the different layers of wounded trees using GC-MS. Mol. Wt., molecular weight; MF, matching factor; RI, retention index.

Compound name	Mol. Wt.	MF	RI	Relative content				
				NL	TL	AL	DA	DL
**Sesquiterpenes**								
8-isopropyl-1,5-dimethyltricyclo[4.4.0.02,7]dec-4-en-3-one	218	678	1687	0.000±0.000	0.000±0.000	0.000±0.000	0.006±0.000	0.000±0.000
5,8a-dimethyl-3-(prop-1-en-2-yl)-1,2,3,7,8,8a-hexahydronaphthalene	202	628	1495	0.000±0.000	0.000±0.000	0.001±0.000	0.000±0.000	0.000±0.000
1,1,7-trimethyl-4-methylenedecahydro-1H-cyclopropa[e]azulen-7-ol	220	763	1576	0.000±0.000	0.000±0.000	0.012±0.000	0.000±0.000	0.000±0.000
2-(4a,8-dimethyl-2,3,4,4a,5,6-hexahydronaphthalen-2-yl)prop-2-en-1-ol	218	674	1727	0.000±0.000	0.000±0.000	0.004±0.000	0.000±0.000	0.000±0.000
2-(6,8-dimethyltricyclo[4.4.0.02,7]dec-8-en-3-yl)propan-2-ol	220	629	1540	0.000±0.000	0.000±0.000	0.002±0.000	0.000±0.000	0.000±0.000
1,1,3a,7-tetramethyl-1a,2,3,3a,4,5,6,7b-octahydro-1H-cyclopropa[a]naphthalene	204	794	1405	0.000±0.000	0.000±0.000	0.004±0.000	0.000±0.000	0.000±0.000
2-((2S,4aS)-4a,8-dimethyl-1,2,3,4,4a,5,6,7-octahydronaphthalen-2-yl)propan-2-ol	222	820	1662	0.000±0.000	0.000±0.000	0.000±0.000	0.000±0.000	0.000±0.000
2-(6,10-dimethylspiro[4.5]dec-6-en-2-yl)propan-2-ol	222	813	1645	0.000±0.000	0.000±0.000	0.001±0.000	0.002±0.000	0.000±0.000
2-(6,8-dimethyltricyclo[4.4.0.02,7]dec-8-en-3-yl)propan-3-ol	223	837	1661	0.000±0.000	0.000±0.000	0.010±0.000	0.011±0.008	0.000±0.000
7-(1-hydroxypropan-2-yl)-1,4a-dimethyl-2,3,4,4a,5,6,7,8-octahydronaphthalen-2-ol	238	667	1901	0.000±0.000	0.000±0.000	0.002±0.000	0.009±0.000	0.000±0.000
4,8a-dimethyl-6-(prop-1-en-2-yl)-1,2,3,5,6,7,8,8a-octahydronaphthalene-2,3-diol	236	687	1878	0.000±0.000	0.003±0.003	0.012±0.000	0.000±0.000	0.000±0.000
7-(2-hydroxypropan-2-yl)-1,4a-dimethyldecahydronaphthalen-1-ol	240	775	1782	0.000±0.000	0.000±0.000	0.008±0.000	0.005±0.001	0.000±0.000
4,4a-dimethyl-6-(prop-1-en-2-yl)-4,4a,5,6,7,8-hexahydronaphthalen-2(3H)-one	218	783	1808	0.000±0.000	0.000±0.000	0.006±0.000	0.000±0.000	0.000±0.000
4,8,8-trimethyldecahydro-1,4-methanoazulene-9-carbaldehyde	220	654	1631	0.000±0.000	0.006±0.002	0.012±0.000	0.000±0.000	0.000±0.000
1,4-dimethyl-7-(propan-2-ylidene)-1,2,3,4,5,6,7,8-octahydroazulene	204	652	1490	0.000±0.000	0.000±0.000	0.007±0.000	0.000±0.000	0.000±0.000
3,8-dimethyl-5-(propan-2-ylidene)-1,2,3,4,5,8a-hexahydro-6H-3a,6-epoxyazulen-6-ol	234	696	1735	0.000±0.000	0.000±0.000	0.010±0.000	0.000±0.000	0.000±0.000
5a,9-dimethyl-3-methylene-3a,4,5,5a,9a,9b-hexahydronaphtho[1,2-b]furan-2(3H)-one	230	734	1895	0.000±0.000	0.000±0.000	0.014±0.000	0.000±0.000	0.000±0.000
6-hydroxy-5a-methyl-3,9-dimethylenedecahydronaphtho[1,2-b]furan-2(3H)-one	248	683	2266	0.000±0.000	0.000±0.000	0.011±0.000	0.020±0.001	0.000±0.000
6-(3-hydroxyprop-1-en-2-yl)-4,8a-dimethyl-3,5,6,7,8,8a-hexahydronaphthalen-2(1H)-one	234	786	1909	0.000±0.000	0.024±0.007	0.025±0.001	0.034±0.003	0.000±0.000

6-(3-hydroxyprop-1-en-2-yl)-4,8a-dimethyl-1,2,3,5,6,7,8,8a-octahydronaphthalen-2-ol	236	795	1933	0.000±0.000	0.008±0.003	0.003±0.006	0.016±0.002	0.000±0.000
7-(2-hydroxypropan-2-yl)-1,4a-dimethyl-4,4a,5,6,7,8-hexahydronaphthalen-2(3H)-one	236	713	1926	0.000±0.000	0.000±0.000	0.033±0.001	0.008±0.006	0.000±0.000
1-hydroxy-4a,5-dimethyl-3-(propan-2-ylidene)-4,4a,5,6-tetrahydronaphthalen-2(3H)-one	232	752	1895	0.000±0.000	0.011±0.003	0.032±0.000	0.022±0.019	0.000±0.000
6,9a-dimethyl-3-methylene-3,3a,4,5,6,6a,9a,9b-octahydroazuleno[4,5-b]furan-2,9-dione	246	671	1943	0.000±0.000	0.000±0.000	0.007±0.000	0.000±0.000	0.000±0.000
2-(4a,8-dimethyl-1,2,3,4,4a,5,6,7-octahydronaphthalen-2-yl)prop-2-en-1-ol	220	689	1745	0.000±0.000	0.000±0.000	0.005±0.000	0.000±0.000	0.000±0.000
4,4a-dimethyl-6-(propan-2-ylidene)-4,4a,5,6,7,8-hexahydronaphthalen-2(3H)-one	218	534	1836	0.000±0.000	0.002±0.000	0.000±0.000	0.008±0.000	0.000±0.000
2-(4a,8-dimethyl-1,2,3,4,4a,5,6,7-octahydronaphthalen-2-yl)propan-2-ol	222	647	1662	0.000±0.000	0.001±0.000	0.000±0.000	0.000±0.000	0.000±0.000
2-(3,8-dimethyl-1,2,3,3a,4,5,6,7-octahydroazulen-5-yl)propan-2-ol	222	565	1667	0.000±0.000	0.005±0.001	0.000±0.000	0.000±0.000	0.000±0.000
3,6-dimethyl-7-(prop-1-en-2-yl)-6-vinylhexahydrobenzofuran-2(3H)-one	234	710	1806	0.000±0.000	0.007±0.001	0.000±0.000	0.000±0.000	0.000±0.000
6-(3-hydroxyprop-1-en-2-yl)-4,8a-dimethyl-1,2,4a,5,6,7,8,8a-octahydronaphthalen-2-ol	236	696	1904	0.000±0.000	0.002±0.004	0.000±0.000	0.015±0.000	0.000±0.000
(1S,4aS,7S,8aS)-7-(2-hydroxypropan-2-yl)-1,4a-dimethyldecahydronaphthalen-1-ol	240	665	1826	0.000±0.000	0.005±0.001	0.000±0.000	0.000±0.000	0.000±0.000
1,4a-dimethyl-7-(prop-1-en-2-yl)-4,4a,5,6,7,8-hexahydronaphthalen-2(3H)-one	218	751	1755	0.000±0.000	0.007±0.003	0.000±0.000	0.000±0.000	0.000±0.000
(Z)-2-(8,8a-dimethyl-3,4,6,7,8,8a-hexahydronaphthalen-2(1H)-ylidene)propanal	218	713	1812	0.000±0.000	0.031±0.008	0.000±0.000	0.000±0.000	0.000±0.000
2-(4a,8-dimethyl-1,2,3,4,4a,5,6,7-octahydronaphthalen-2-yl)prop-2-en-1-ol	220	608	1745	0.000±0.000	0.001±0.001	0.000±0.000	0.018±0.001	0.000±0.000
2-(4a,8-dimethyl-2,3,4,4a,5,6-hexahydronaphthalen-2-yl)prop-2-en-1-ol	218	712	1727	0.000±0.000	0.004±0.001	0.000±0.000	0.000±0.000	0.000±0.000
(1S,4S)-1,4-dimethyl-7-(propan-2-ylidene)-1,2,3,4,5,6,7,8-octahydroazulene	204	747	1490	0.000±0.000	0.000±0.000	0.000±0.000	0.010±0.000	0.000±0.000
1,1,7,7a-tetramethyl-1a,2,4,5,6,7,7a,7b-octahydro-1H-cyclopropa[a]naphthalene	204	781	1453	0.000±0.000	0.000±0.000	0.000±0.000	0.000±0.000	0.000±0.000
2-(3,8-dimethyl-1,2,3,4,5,6,7,8-octahydroazulen-5-yl)propan-2-ol	222	777	1596	0.000±0.000	0.000±0.000	0.000±0.000	0.007±0.009	0.000±0.000
(4aS,5R)-4a,5-dimethyl-3-(propan-2-ylidene)-4,4a,5,6,7,8-hexahydronaphthalen-2(3H)-one	218	777	1817	0.000±0.000	0.000±0.000	0.000±0.000	0.010±0.008	0.000±0.000
(3aR,4R,7R)-1,4,9,9-tetramethyl-5,6,7,8-tetrahydro-3H-3a,7-methanoazulen-2(4H)-one	218	805	1687	0.000±0.000	0.000±0.000	0.000±0.000	0.005±0.009	0.000±0.000
4a,9-dimethyl-7-(prop-1-en-2-yl)-3a,4,4a,5,6,7,8,9,9a-octahydronaphtho[2,3-d][1,3]dioxol-2-one	262	530	1978	0.000±0.000	0.000±0.000	0.000±0.000	0.006±0.000	0.000±0.000
4,4,6b-trimethyl-2-(prop-1-en-2-yl)-4,5,5a,6,6a,6b-hexahydro-2H-cyclopropa[g]benzofuran	218	694	1438	0.000±0.000	0.000±0.000	0.000±0.000	0.054±0.002	0.000±0.000
2a,5a,9-trimethyl-2,2a,5,5a,6,7,8,9b-octahydro-4H-naphtho[1,2-b]oxireno[2,3-c]furan	234	825	1957	0.000±0.000	0.024±0.003	0.000±0.000	0.023±0.002	0.000±0.000
3,6,9-trimethylenedecahydroazuleno[4,5-b]furan-2(3H)-one	230	655	2007	0.000±0.000	0.004±0.001	0.000±0.000	0.000±0.000	0.000±0.000
1,2,3a,6-tetramethyl-4,5,5a,6,7,8-hexahydrocyclopenta[c]pentalen-3(3aH)-one	218	677	1623	0.000±0.000	0.005±0.008	0.000±0.000	0.000±0.000	0.000±0.000
1,4,9,9-tetramethyloctahydro-4,7-(epoxymethano)azulen-3a(1H)-ol	238	561	1674	0.000±0.000	0.000±0.000	0.002±0.000	0.000±0.000	0.000±0.000

1-methyl-7-(3-oxobutyl)-4-(propan-2-ylidene)bicyclo[4.1.0]heptan-3-one	234	662	1844	0.000±0.000	0.000±0.000	0.006±0.000	0.000±0.000	0.000±0.000
5,8-dihydroxy-4a-methyl-4,4a,4b,5,6,7,8,8a,9,10-decahydrophenanthren-2(3H)-one	250	779	2115	0.000±0.000	0.005±0.003	0.037±0.001	0.016±0.004	0.000±0.000
(Z)-1,2,3a,7-tetramethyl-1,2,3,3a,4,5,7,8-octahydro-6H-cyclopenta[8]annulen-6-one	220	683	1660	0.000±0.000	0.000±0.000	0.036±0.001	0.000±0.000	0.000±0.000
1-(4-hydroxy-4,7,7-trimethyl-3-(prop-1-en-2-yl)cyclohept-1-en-1-yl)ethan-1-one	236	641	1720	0.000±0.000	0.000±0.000	0.007±0.000	0.000±0.000	0.000±0.000
2,4a,5,8a-tetramethyl-1,2,3,4,4a,7,8,8a-octahydronaphthalen-1-yl acetate	250	621	1709	0.000±0.000	0.000±0.000	0.007±0.000	0.000±0.000	0.000±0.000
6a-hydroxy-6,9a-dimethyl-3-methylenedecahydroazuleno[4,5-b]furan-2,9-dione	264	573	2112	0.000±0.000	0.004±0.002	0.000±0.000	0.000±0.000	0.000±0.000
methyl 2,5,5,8a-tetramethyl-3-oxo-3,4,4a,5,6,7,8,8a-octahydronaphthalene-1-carboxylate	264	577	1909	0.000±0.000	0.002±0.000	0.000±0.000	0.000±0.000	0.000±0.000
**Total**				0.000±0.000	0.194±0.057	0.445±0.004	0.351±0.028	0.000±0.000
**Chromone**								
2-phenethyl-4H-chromen-4-one	250	799	2346	0.000±0.000	0.001±0.000	0.002±0.000	0.002±0.000	0.000±0.000
6-methoxy-2-phenethyl-4H-chromen-4-one	280	890	2613	0.000±0.000	0.000±0.000	0.005±0.000	0.014±0.000	0.000±0.000
2-(4-methoxyphenethyl)-4H-chromen-4-one	280	835	2545	0.000±0.000	0.001±0.001	0.004±0.000	0.000±0.000	0.000±0.000
6,7-dimethoxy-2-phenethyl-4H-chromen-4-one	310	875	2891	0.000±0.000	0.010±0.003	0.032±0.000	0.038±0.002	0.000±0.000
6,7-dimethoxy-2-(4-methoxyphenethyl)-4H-chromen-4-one	340	872	3145	0.000±0.000	0.011±0.003	0.034±0.000	0.032±0.001	0.000±0.000
**Total**				0.000±0.000	0.023±0.007	0.077±0.000	0.086±0.002	0.000±0.000

Table S2. Data pre-processing statistics and quality control of high-throughput fungal sequencing diversity in different layers of agarwood.

Sample name	Raw reads	Clean Reads	Base (nt)	Average Length (nt)	Q20	GC%	Effective (%)
DL1	83499	80127	19682931	245	82.06	51.33	95.96
DL2	81636	80162	20945823	261	81.71	48.11	98.19
DL3	84837	80331	19641390	244	81.25	51.71	94.69
DA1	81228	80078	19766309	246	84.55	50.58	98.58
DA2	81663	80076	18470974	230	84.14	48.51	98.06
DA3	81165	80084	18870842	235	83.94	48.47	98.67
AL1	82739	80335	18870256	234	82.42	48.69	97.09
AL2	80612	80122	18343223	228	87.79	48.38	99.39
AL3	82174	80084	17895897	223	81.48	48.52	97.46
TL1	80910	80207	18758865	233	84.41	53.57	99.13
TL2	81295	80125	18667004	232	83.47	51.52	98.56
TL3	81226	80182	18503062	230	84.88	52.32	98.71
NL1	80939	80073	18827915	235	82.9	50.18	98.93
NL2	81710	80218	19141925	238	83.58	49.2	98.17
NL3	80852	80055	18381746	229	80.49	49.04	99.01

Table S3. Tukey analysis of significant difference of diversity index among different groups.

	Observed species	ACE	Chao 1	PD whole tree	Shannon
DA-AL	0.1970	0.2123	0.1374	0.9517	0.1258
DL-AL	0.0001	0.0001	0.0001	0.0061	0.0008
NL-AL	0.0316	0.0550	0.0337	0.0118	0.0199
TL-AL	0.9033	0.9023	0.8766	0.9985	0.8853
DL-DA	0.0018	0.0017	0.0012	0.0175	0.0396
NL-DA	0.7494	0.8911	0.8848	0.0346	0.7538
TL-DA	0.5744	0.6057	0.4781	0.8592	0.4361
NL-DL	0.0101	0.0061	0.0044	0.9908	0.2392
TL-DL	0.0003	0.0003	0.0002	0.0041	0.0028
TL-NL	0.1197	0.2033	0.1418	0.0078	0.0819

Table S4. Sesquiterpene and chromone compounds identified in *P. rubrigenum*-treated *A. sinensis* stems using GC-MS. Mol. Wt., molecular weight; MF, matching factor; CAS No., library CAS number; RI, retention index.

Compound name	Mol. Wt.	MF	RI	Relative content (%)							
				0d	5d	10d	15d	20d	25d	30d	CK 30d
**Sesquiterpenes**											
Dehydrosaussurea lactone	232	501	1838	0.000±0.000	0.000±0.000	0.000±0.000	0.000±0.000	0.000±0.000	0.000±0.000	0.001±0.000	0.000±0.000
Longifolenaldehyde	220	585	1631	0.000±0.000	0.000±0.000	0.000±0.000	0.000±0.000	0.000±0.000	0.237±0.030	0.174±0.005	0.000±0.000
7-isopropyl-1,4-dimethylazulen-2-ol	214	701	1934	0.000±0.000	0.325±0.012	0.240±0.004	0.000±0.000	0.000±0.000	0.000±0.000	0.000±0.000	0.000±0.000
α-bulnesene	204	505	1505	0.000±0.000	0.000±0.000	0.059±0.005	0.000±0.000	0.071±0.007	0.000±0.000	0.000±0.000	0.000±0.000
2a,5a,9-trimethyl-2,2a,5,5a,6,7,8,9b-octahydro-4H-naphtho[1,2-b]oxireno[2,3-c]furan	234	701	1957	0.000±0.000	0.000±0.000	0.111±0.014	0.171±0.115	0.687±0.071	0.455±0.047	0.547±0.026	0.000±0.000
3,6,9-trimethylenedecahydroazuleno[4,5-b]furan-2(3H)-one	230	624	2007	0.000±0.000	0.000±0.000	0.148±0.014	0.000±0.000	0.000±0.000	0.000±0.000	0.570±0.035	0.000±0.000
6-hydroxy-5a-methyl-3,9-dimethylenedecahydronaphtho[1,2-b]furan-2(3H)-one	248	713	2266	0.000±0.000	0.000±0.000	0.245±0.013	0.161±0.082	0.387±0.015	0.284±0.039	0.230±0.008	0.000±0.000
1,4,9,9-tetramethyloctahydro-4,7-(epoxymethano)azulen-3a(1H)-ol	238	499	1674	0.000±0.000	0.000±0.000	0.000±0.000	0.111±0.036	0.000±0.000	0.000±0.000	0.000±0.000	0.000±0.000
2-(4a-methyl-8-methylenedecahydronaphthalen-2-yl)prop-2-en-1-ol	220	630	1778	0.000±0.000	0.000±0.000	0.000±0.000	0.051±0.028	0.410±0.025	0.420±0.039	0.000±0.000	0.000±0.000

5,8-dihydroxy-4a-methyl-4,4a,4b,5,6,7,8,8a,9,10-decahydrophenanthren-2(3H)-one	250	694	2115	0.000±0.000	0.000±0.000	0.000±0.000	0.341±0.145	0.533±0.034	0.626±0.069	0.590±0.039	0.000±0.000
Aromadendrene epoxide	220	717	1462	0.000±0.000	0.000±0.000	0.000±0.000	0.000±0.000	0.000±0.000	0.000±0.000	0.516±0.009	0.000±0.000
Isoaromadendrene epoxide	220	726	1589	0.000±0.000	0.000±0.000	0.000±0.000	0.000±0.000	0.000±0.000	0.000±0.000	0.193±0.095	0.000±0.000
(-)-Globulol	222	621	1591	0.000±0.000	0.000±0.000	0.000±0.000	0.000±0.000	0.000±0.000	0.000±0.000	0.095±0.022	0.000±0.000
(-)-α-Selinene	204	714	1494	0.000±0.000	0.000±0.000	0.000±0.000	0.000±0.000	0.000±0.000	0.000±0.000	0.434±0.211	0.000±0.000
2-((2R,4aR,8aR)-4a,8-dimethyl-1,2,3,4,4a,5,6,8a-octahydronaphthalen-2-yl)prop-2-en-1-ol	220	807	1778	0.000±0.000	0.000±0.000	0.000±0.000	0.000±0.000	0.000±0.000	0.194±0.019	0.388±0.012	0.000±0.000
6-(3-hydroxyprop-1-en-2-yl)-4,8a-dimethyl-3,5,6,7,8,8a-hexahydronaphthalen-2(1H)-one	234	755	1909	0.000±0.000	0.000±0.000	0.000±0.000	0.000±0.000	0.098±0.038	0.000±0.000	0.464±0.052	0.000±0.000
Velleral	232	603	1817	0.000±0.000	0.000±0.000	0.000±0.000	0.000±0.000	0.258±0.019	0.272±0.026	0.188±0.011	0.000±0.000
2-(4a-methyl-8-methylene-1,4,4a,5,6,7,8,8a-octahydronaphthalen-2-yl)propan-1-ol	220	562	1723	0.000±0.000	0.000±0.000	0.000±0.000	0.000±0.000	0.000±0.000	0.053±0.020	0.000±0.000	0.000±0.000
(4α,5β,6α,7α,10α)-1-Aromadendrene	204	655	1447	0.000±0.000	0.000±0.000	0.000±0.000	0.000±0.000	0.173±0.039	0.000±0.000	0.000±0.000	0.000±0.000
isointermedeol	222	592	1660	0.000±0.000	0.000±0.000	0.000±0.000	0.000±0.000	0.384±0.112	0.000±0.000	0.000±0.000	0.000±0.000
(Z)-α-Santalol	220	821	1669	0.000±0.000	0.000±0.000	0.000±0.000	0.000±0.000	0.673±0.030	0.819±0.085	0.979±0.043	0.000±0.000

(E)-5-(2,3-dimethyltricyclo[2.2.1.02,6]heptan-3-yl)-2-methylpent-2-enal	218	617	1679	0.000±0.000	0.000±0.000	0.000±0.000	0.000±0.000	0.000±0.000	0.000±0.000	0.164±0.008	0.000±0.000
5a,9,9-trimethyloctahydro-2H,4H-benzo[d]cyclopropa[c]oxepine-2,4-dione	236	574	1861	0.000±0.000	0.000±0.000	0.000±0.000	0.099±0.095	0.000±0.000	0.000±0.000	0.000±0.000	0.000±0.000
(-)-β-elemene	204	660	1391	0.000±0.000	0.000±0.000	0.000±0.000	0.000±0.000	0.000±0.000	0.000±0.000	0.039±0.009	0.000±0.000
2-isopropyl-4-methylcyclohex-3-en-1-yl (E)-2-methylbut-2-enoate	236	580	1545	0.000±0.000	0.000±0.000	0.000±0.000	0.089±0.053	0.000±0.000	0.000±0.000	0.000±0.000	0.000±0.000
elemol	222	527	1549	0.000±0.000	0.000±0.000	0.000±0.000	0.000±0.000	0.000±0.000	0.184±0.021	0.000±0.000	0.000±0.000
dihydropyrocurzerenone	214	757	1861	0.000±0.000	0.720±0.014	0.526±0.038	0.344±0.123	0.000±0.000	0.264±0.065	0.000±0.000	0.000±0.000
Total				0.000±0.000	1.045±0.012	1.328±0.037	1.358±0.271	3.673±0.170	3.809±0.400	5.572±0.225	0.000±0.000
**Chromone**											
2-(4-methoxyphenethyl)-4H-chromen-4-one	280	743	2545	0.000±0.000	0.000±0.000	0.000±0.000	0.000±0.000	0.105±0.029	0.245±0.174	0.260±0.180	0.000±0.000

Table S5. Sesquiterpene compounds identified in *P. rubrigenum*-treated *A. sinensis* calli using GC-MS. Mol. Wt., molecular weight; MF, matching factor; CAS No., library CAS number; RI, retention index, n=3.

Compound	CAS Num.	Match factor	RT	RI	0 day	3 day	18 day	p-value (0d vs. 3day)	p-value (0d vs. 18day)
δ-guaiene	3691-11-0	95.15248	25.2430	1508.604	15926.59±2060.64	51638.86±41800.24	208693.80±26738.96	0.138452	0.003056
α-copaene	3856-25-5	61.98009	25.9186	1536.869	879.69±294.38	10890.07±7070.17	38455.85±7704.07	0.066740	0.006812
α-guaiene	3691-12-1	97.80873	23.5702	1441.833	2388.78±1165.68	9697.91±8471.84	117001.30±46903.98	0.136290	0.025733
nootkatene	5090-61-9	77.55274	25.9186	1536.869	879.89±294.50	10933.67±7046.91	38790.20±7274.857	0.065918	0.005975

Table S6. Relative TMT protein expression quantities of the proteins involved in sesquiterpene biosynthesis. Data are shown as mean ± SD, compared to 0 day group. P value is evaluated by T-test. n=3.

Gene Number	Gene symbol	0d	3d	18d	p-value (0d vs. 3day)	p-value (0d vs. 18day)
evm.model.Scaffold8.236	AACT1	0.700±0.018	0.681±0.008	0.944±0.060	0.106923	0.006936
evm.model.Scaffold19.270	AACT2	0.940±0.008	0.953±0.042	0.920±0.033	0.321306	0.201382
evm.model.Scaffold3.319	HMGS	0.646±0.003	0.703±0.060	0.908±0.083	0.119698	0.015648
evm.model.Scaffold283.29	HMGR	0.790±0.058	0.747±0.003	0.956±0.042	0.163547	0.009502
evm.model.Scaffold68.185	MVK	0.866±0.016	0.901±0.067	0.988±0.014	0.236010	0.000306
evm.model.Scaffold223.14	PMK	0.750±0.014	0.755±0.035	0.970±0.031	0.410184	0.001084
evm.model.Scaffold270.65	MDC	0.802±0.009	0.792±0.029	0.969±0.033	0.313184	0.004386
evm.model.Scaffold160.11	DXS1	0.851±0.020	0.924±0.081	0.775±0.034	0.126840	0.020101
evm.model.Scaffold23.83	DXS3	0.827±0.077	0.804±0.071	0.902±0.117	0.362802	0.206841
evm.model.Scaffold181.5	DXR	0.950±0.047	0.872±0.007	0.792±0.018	0.049618	0.009015
evm.model.Scaffold1.714	MCT	0.897±0.012	0.933±0.073	0.876±0.030	0.245630	0.173983
evm.model.Scaffold15.74	CMK	0.936±0.011	0.906±0.083	0.855±0.013	0.299242	0.000627
evm.model.Scaffold3.223	MCS	0.951±0.031	0.940±0.065	0.886±0.025	0.401526	0.025003
evm.model.Scaffold4.152	HDS	0.982±0.017	0.964±0.035	0.818±0.027	0.243180	0.000995
evm.model.Scaffold6.446	HDR1	0.842±0.032	0.959±0.047	0.907±0.047	0.014493	0.062803
evm.model.Scaffold2.705	HDR2	0.982±0.020	0.828±0.102	0.611±0.022	0.058065	0.000015
evm.model.Scaffold448.7	IPPI	0.687±0.019	0.748±0.015	0.981±0.023	0.006763	0.000047
evm.model.Scaffold125.4	FPS	0.646±0.019	0.599±0.064	0.934±0.090	0.165431	0.013328
evm.model.Scaffold11.100	SesTPS1	0.511±0.116	0.468±0.019	0.818±0.172	0.294664	0.035417
evm.model.Scaffold11.101	SesTPS2	0.196±0.006	0.210±0.037	0.705±0.256	0.284867	0.037327
evm.model.Scaffold70.77	SesTPS3	0.687±0.019	0.598±0.021	0.846±0.135	0.002655	0.087695
evm.model.Scaffold49.131	SesTPS4	0.737±0.005	0.935±0.066	0.663±0.016	0.017205	0.004873

Table S7. Relative TMT protein expression quantities of the proteins involved in transcription factor. n=3. Data are shown as mean ± SD, compared to 0 day group. P value is evaluated by T-test.

Gene number	Gene symbol	0d	3d	18d	p-value (0d vs. 3day)	p-value (0d vs. 18day)
**bHLH**						
evm.model.Scaffold147.29	bHLH3	0.929±0.038	0.951±0.045	0.947±0.050	0.551584	0.650949
evm.model.Scaffold235.11	bHLH128	0.964±0.032	0.951±0.031	0.795±0.025	0.648852	0.002418
evm.model.Scaffold31.108	BIM2	0.978±0.025	0.922±0.054	0.707±0.065	0.204124	0.010601
evm.model.Scaffold32.21	MYC2	0.814±0.163	0.621±0.020	0.607±0.094	0.176193	0.147234
evm.model.Scaffold401.3	bHLH66	0.937±0.062	0.948±0.030	0.742±0.084	0.799980	0.035869
evm.model.Scaffold8.8	UNE12	0.898±0.089	0.925±0.040	0.840±0.017	0.672495	0.370737
evm.model.Scaffold61.76	bHLH169	0.935±0.053	0.878±0.041	0.945±0.049	0.215735	0.833096
**ERF**						
evm.model.Scaffold307.28	ERF114	0.817±0.161	0.685±0.147	0.775±0.110	0.352657	0.727864
evm.model.Scaffold74.54	ERF113	0.838±0.056	0.833±0.089	0.948±0.045	0.936569	0.060995
evm.model.Scaffold9.119	ERF113	0.893±0.044	0.945±0.050	0.833±0.030	0.245424	0.135197
evm.model.Scaffold137.11	ABR1-like	0.491±0.046	0.442±0.049	0.754±0.257	0.282725	0.214938
**WRKY**						
evm.model.Scaffold19.164	WRKY6	0.826±0.083	0.766±0.156	0.963±0.048	0.596391	0.083683
evm.model.Scaffold369.3	WRKY24	0.782±0.046	0.788±0.173	0.889±0.142	0.962288	0.321071
evm.model.Scaffold49.21	WRKY31	0.797±0.010	0.796±0.172	0.927±0.112	0.995914	0.179281
evm.model.Scaffold6.65	WRKY9	0.883±0.062	0.864±0.043	0.885±0.113	0.690666	0.974102
**MYB**						
evm.model.Scaffold12.60	MYBS3	0.976±0.027	0.911±0.020	0.846±0.077	0.034486	0.087326
evm.model.Scaffold135.18	ARR2	0.667±0.014	0.771±0.041	0.942±0.054	0.037533	0.008889
evm.model.Scaffold20.252	DIVARICATA	0.749±0.138	0.808±0.192	0.782±0.059	0.690355	0.733971

evm.model.Scaffold25.241	SWI3C	0.673±0.284	0.524±0.028	0.479±0.026	0.459484	0.357803
evm.model.Scaffold274.23	TRB2-2	0.938±0.087	0.875±0.090	0.820±0.033	0.439071	0.130811
evm.model.Scaffold306.42	SWR1-4	0.942±0.028	0.949±0.054	0.944±0.038	0.845524	0.931647
evm.model.Scaffold3.515	MYB1R1-2	0.821±0.082	0.816±0.170	0.920±0.063	0.969787	0.178714
evm.model.Scaffold38.14	ALY2	0.861±0.020	0.837±0.077	0.982±0.017	0.647062	0.001547
evm.model.Scaffold446.23	SRM1	0.921±0.068	0.863±0.022	0.877±0.024	0.270443	0.375851
evm.model.Scaffold60.44	GAMYB	0.922±0.067	0.817±0.051	0.837±0.056	0.101718	0.170454
evm.model.Scaffold62.84	SWI3D	0.958±0.031	0.896±0.099	0.862±0.035	0.394647	0.024925
evm.model.Scaffold6.610	SRM1	0.992±0.007	0.945±0.019	0.915±0.069	0.036236	0.189352
evm.model.Scaffold67.8	TRB4	0.947±0.054	0.922±0.023	0.913±0.050	0.525474	0.470894
evm.model.Scaffold74.6	SWI3A	0.770±0.031	0.725±0.038	0.836±0.143	0.187630	0.512058
**bZIP**						
evm.model.Scaffold252.12	TGA2	0.685±0.028	0.752±0.049	0.869±0.118	0.124534	0.108030
evm.model.Scaffold257.55	HY5-X1	0.954±0.031	0.940±0.065	0.792±0.068	0.765210	0.037306
evm.model.Scaffold2.64	VIP1	0.973±0.027	0.905±0.018	0.758±0.063	0.028452	0.016170
evm.model.Scaffold29.250	bZIP16	0.881±0.071	0.907±0.082	0.886±0.025	0.698031	0.911346
evm.model.Scaffold79.16	RF2b	0.790±0.003	0.901±0.097	0.701±0.109	0.185726	0.295927
evm.model.Scaffold1.123	GBF1	0.883±0.059	0.915±0.058	0.932±0.059	0.543557	0.365435
evm.model.Scaffold178.27	ABI5	0.878±0.059	0.812±0.168	0.823±0.150	0.577114	0.602838
evm.model.Scaffold18.16	VIP1	0.820±0.014	0.834±0.038	0.934±0.061	0.609563	0.076057
evm.model.Scaffold18.375	HBP-1b	0.822±0.100	0.799±0.176	0.686±0.092	0.858464	0.157425
evm.model.Scaffold2.254	ABF2	0.891±0.101	0.727±0.019	0.757±0.112	0.103107	0.202878
**NAC**						
evm.model.Scaffold15.182	NAC8	0.936±0.055	0.903±0.022	0.879±0.026	0.416004	0.205571
evm.model.Scaffold175.42	NAC62	0.960±0.036	0.872±0.058	0.782±0.072	0.101948	0.031598
evm.model.Scaffold19.309	NAC17	0.610±0.015	0.419±0.060	0.910±0.102	0.026326	0.033672

evm.model.Scaffold229.28	NAC72	0.914±0.059	0.808±0.021	0.940±0.089	0.077567	0.699607
evm.model.Scaffold25.232	NAC5	0.819±0.068	0.825±0.110	0.916±0.076	0.935760	0.172721
**MADS**						
evm.model.Scaffold2.650	MADS	0.946±0.065	0.596±0.040	0.581±0.021	0.002948	0.006118
**DOF**						
evm.model.Scaffold13.356	DOF4.6	0.849±0.045	0.878±0.106	0.689±0.083	0.696171	0.057428
**HD**						
evm.model.Scaffold21.220	KNAT3	0.903±0.008	0.923±0.103	0.873±0.046	0.770304	0.383749
evm.model.Scaffold30.134	WOX13	0.951±0.043	0.833±0.058	0.795±0.127	0.050540	0.157409
evm.model.Scaffold38.162	RLT1	0.990±0.014	0.920±0.018	0.939±0.022	0.007345	0.035568
evm.model.Scaffold51.18	BLH1	0.924±0.066	0.910±0.022	0.846±0.040	0.768130	0.174399
**TCP**						
evm.model.Scaffold10.275	TCP20	0.885±0.095	0.882±0.120	0.704±0.127	0.973029	0.123209
evm.model.Scaffold18.264	TCP23	0.676±0.063	0.799±0.107	0.868±0.118	0.180055	0.087247
evm.model.Scaffold6.621	TCP21	0.875±0.113	0.882±0.096	0.797±0.068	0.936660	0.375925
**SBP**						
evm.model.Scaffold306.21	SBP1	0.969±0.027	0.977±0.020	0.967±0.029	0.691901	0.946617
**GRAS**						
evm.model.Scaffold44.37	SCL1	0.958±0.017	0.959±0.057	0.831±0.027	0.969754	0.004121
evm.model.Scaffold52.21	SCL33	0.743±0.023	0.654±0.037	0.809±0.166	0.031120	0.562825
evm.model.Scaffold19.245	SCL13	0.957±0.037	0.906±0.046	0.891±0.067	0.211387	0.227482
evm.model.Scaffold20.291	SCL30	0.975±0.025	0.852±0.099	0.765±0.020	0.157683	0.000465
evm.model.Scaffold20.292	SCL14	0.720±0.040	0.771±0.045	0.914±0.098	0.221672	0.060587

Table S8. Relative protein phosphorylation quantities of the proteins involved in transcription factor. Data are shown as mean ± SD, compared to 0 day group. P value is evaluated by T-test. n=3.

Gene number	Gene symbol	0d	3d	18d	p-value (0d vs. 3day)	p-value (0d vs. 18day)
**bHLH**						
evm.model.Scaffold109.64	bHLH122	0.561±0.251	0.352±0.163	0.802±0.258	0.151815	0.155343
evm.model.Scaffold147.29	bHLH3	0.858±0.216	0.840±0.167	0.658±0.211	0.458116	0.156971
evm.model.Scaffold19.32	bHLH130	0.711±0.342	0.268±0.080	0.359±0.003	0.073879	0.108037
evm.model.Scaffold229.30	SPT	0.133±0.058	0.766±0.382	0.192±0.104	0.049997	0.226910
evm.model.Scaffold235.11	bHLH128	0.489±0.253	0.649±0.338	0.179±0.089	0.275590	0.079138
evm.model.Scaffold2.401	bHLH93	0.745±0.226	0.516±0.186	0.321±0.119	0.124713	0.031382
evm.model.Scaffold31.108	BIM2	0.547±0.060	0.726±0.240	0.465±0.089	0.161986	0.122576
evm.model.Scaffold32.21	MYC2	0.711±0.404	0.849±0.118	0.418±0.265	0.308815	0.180689
evm.model.Scaffold59.32	bHLH28	0.598±0.528	0.316±0.366	0.133±0.085	0.247328	0.132479
evm.model.Scaffold70.20	BIM1	0.954±0.066	0.726±0.140	0.818±0.146	0.044249	0.122435
evm.model.Scaffold7.142	bHLH13	0.354±0.172	0.595±0.356	0.387±0.342	0.184851	0.444863
evm.model.Scaffold8.8	UNE12	0.900±0.160	0.394±0.352	0.132±0.228	0.057286	0.005768
evm.model.Scaffold26.94	bHLH156-1	0.692±0.141	0.559±0.356	0.113±0.196	0.349652	0.008627
evm.model.Scaffold51.17	bHLH156-2	0.331±0.106	0.606±0.347	0.464±0.072	0.151429	0.077648
evm.model.Scaffold61.76	bHLH169	0.000±0.000	0.110±0.101	0.706±0.255	0.100590	0.020409
**ERF**						
evm.model.Scaffold12.183	RAP2-12	0.872±0.127	0.320±0.105	0.212±0.212	0.002446	0.007825
evm.model.Scaffold1.344	ANT	0.881±0.145	0.794±0.164	0.211±0.169	0.265599	0.003412
evm.model.Scaffold24.107	ERF12	0.549±0.507	0.686±0.096	0.000±0.000	0.344554	0.100794
evm.model.Scaffold267.44	ERF027	0.748±0.227	0.289±0.111	0.509±0.124	0.026906	0.102681
evm.model.Scaffold321.39	ERF7	0.749±0.072	0.824±0.155	0.000±0.000	0.254270	0.001523

evm.model.Scaffold35.81	ERF003	0.556±0.509	0.259±0.232	0.135±0.235	0.214840	0.144967
evm.model.Scaffold74.54	ERF113	0.743±0.137	0.532±0.037	0.867±0.122	0.054145	0.152598
**WRKY**						
evm.model.Scaffold10.342	WRKY32	0.842±0.148	0.896±0.037	0.641±0.126	0.298504	0.074572
evm.model.Scaffold111.13	WRKY20	0.277±0.246	0.636±0.333	0.094±0.082	0.106512	0.163659
evm.model.Scaffold1.247	WRKY15	0.419±0.086	0.567±0.211	0.958±0.045	0.176384	0.001141
evm.model.Scaffold129.9	WRKY65	0.569±0.089	0.358±0.014	0.845±0.205	0.026015	0.065579
evm.model.Scaffold13.282	WRKY30	0.401±0.115	0.334±0.159	0.493±0.445	0.295821	0.379376
evm.model.Scaffold155.37	WRKY62	0.353±0.027	0.425±0.199	0.856±0.205	0.298418	0.010386
evm.model.Scaffold1.584	WRKY28	0.374±0.325	0.506±0.500	0.444±0.417	0.361962	0.415700
evm.model.Scaffold18.188	WRKY14	0.267±0.046	0.377±0.139	0.607±0.349	0.152009	0.115971
evm.model.Scaffold19.164	WRKY6	0.592±0.065	0.501±0.432	0.544±0.212	0.375393	0.368874
evm.model.Scaffold229.22	WRKY7	0.420±0.069	0.756±0.113	0.719±0.251	0.008783	0.083709
evm.model.Scaffold30.90	WRKY2	0.736±0.092	0.734±0.098	0.800±0.226	0.492547	0.340160
evm.model.Scaffold31.15	WRKY4	0.706±0.225	0.289±0.104	0.862±0.168	0.033017	0.197443
evm.model.Scaffold31.48	WRKY57	0.776±0.203	0.646±0.133	0.344±0.310	0.205945	0.062119
evm.model.Scaffold369.3	WRKY24	0.367±0.094	0.432±0.144	0.803±0.177	0.277562	0.015848
evm.model.Scaffold47.119	WRKY44	0.436±0.466	0.226±0.070	0.909±0.093	0.258683	0.108733
evm.model.Scaffold49.21	WRKY31	0.486±0.036	0.505±0.181	0.828±0.202	0.437853	0.047381
evm.model.Scaffold6.65	WRKY9	0.357±0.270	0.170±0.147	0.787±0.251	0.183544	0.056809
evm.model.Scaffold7.283	WRKY23	0.946±0.066	0.000±0.000	0.000±0.000	0.000809	0.000809
evm.model.Scaffold8.205	WRKY1	0.731±0.135	0.513±0.096	0.696±0.265	0.045751	0.426346
evm.model.Scaffold8.125	WRKY42	0.131±0.005	0.061±0.106	0.732±0.308	0.186231	0.038626
**bZIP**						
evm.model.Scaffold178.27	ABI5	0.751±0.276	0.514±0.128	0.161±0.050	0.137713	0.030864
evm.model.Scaffold18.16	VIP1	0.622±0.325	0.492±0.461	0.368±0.224	0.355514	0.167209

evm.model.Scaffold18.375	HBP-1b	0.394±0.179	0.333±0.079	0.730±0.238	0.315006	0.063894
evm.model.Scaffold206.21	CPRF1	0.771±0.293	0.725±0.137	0.505±0.123	0.412618	0.126590
evm.model.Scaffold21.302	bZIP9	0.499±0.401	0.432±0.493	0.116±0.122	0.431724	0.118684
evm.model.Scaffold2.254	ABF2	0.889±0.097	0.744±0.127	0.710±0.122	0.098328	0.060251
evm.model.Scaffold235.68	PosF21	0.634±0.261	0.758±0.221	0.838±0.099	0.282437	0.154028
evm.model.Scaffold23.68	HY5-X2	0.872±0.024	0.980±0.017	0.614±0.069	0.002074	0.007642
evm.model.Scaffold25.139	TGA7	0.604±0.349	0.000±0.000	0.101±0.174	0.047788	0.056620
evm.model.Scaffold252.12	TGA2	0.546±0.156	0.770±0.200	0.606±0.198	0.102538	0.351797
evm.model.Scaffold257.55	HY5-X1	0.784±0.196	0.745±0.064	0.425±0.078	0.384785	0.035341
evm.model.Scaffold2.64	VIP1	0.880±0.048	0.928±0.086	0.244±0.031	0.225058	0.000062
evm.model.Scaffold28.146	GBF4	0.882±0.111	0.794±0.060	0.522±0.139	0.155217	0.013353
evm.model.Scaffold28.63	bZIP23	0.638±0.119	0.816±0.161	0.497±0.089	0.101803	0.090379
evm.model.Scaffold29.250	bZIP16	0.727±0.168	0.683±0.275	0.770±0.123	0.413228	0.371283
evm.model.Scaffold294.26	VIP1	0.485±0.099	0.715±0.275	0.249±0.012	0.140926	0.025904
evm.model.Scaffold294.8	GBF4	0.811±0.168	0.581±0.074	0.624±0.099	0.063212	0.093946
evm.model.Scaffold4.147	ABI5-2	0.918±0.072	0.627±0.392	0.876±0.039	0.163438	0.221636
evm.model.Scaffold4.222	CPRF2	0.283±0.055	0.346±0.186	0.810±0.242	0.313214	0.028659
evm.model.Scaffold6.197	RF2	0.839±0.224	0.782±0.235	0.246±0.039	0.388114	0.020482
evm.model.Scaffold6.343	ABI5-7	0.800±0.333	0.000±0.000	0.000±0.000	0.026552	0.026552
evm.model.Scaffold64.58	TGA9	0.126±0.022	0.000±0.000	0.817±0.255	0.004839	0.020919
evm.model.Scaffold7.106	GPRF-1	0.954±0.040	0.650±0.147	0.193±0.334	0.030510	0.028262
evm.model.Scaffold79.16	RF2b	0.663±0.345	0.605±0.399	0.364±0.228	0.429712	0.144498
evm.model.Scaffold8.5	CPRF2	0.651±0.096	0.672±0.163	0.877±0.136	0.428251	0.042810
**MYB**						
evm.model.Scaffold74.6	SWI3A	0.960±0.035	0.839±0.125	0.609±0.072	0.116342	0.002607
evm.model.Scaffold24.267	SWI3B	0.751±0.222	0.584±0.084	0.655±0.056	0.161166	0.268272

evm.model.Scaffold25.241	SWI3C	0.861±0.040	0.755±0.215	0.698±0.095	0.243394	0.040155
evm.model.Scaffold62.84	SWI3D	0.884±0.055	0.842±0.149	0.718±0.126	0.341581	0.068066
evm.model.Scaffold8.383	At1g14600	0.027±0.027	0.241±0.272	0.764±0.228	0.153196	0.014537
evm.model.Scaffold98.8	PHL6	0.020±0.017	0.145±0.160	0.946±0.091	0.153633	0.001231
evm.model.Scaffold224.56	EFM	0.000±0.000	0.393±0.533	0.287±0.264	0.165011	0.100068
evm.model.Scaffold85.41	MYB25	0.162±0.281	0.617±0.539	0.628±0.062	0.142840	0.048219
evm.model.Scaffold12.60	MYBS3	0.487±0.018	0.611±0.339	0.450±0.061	0.294456	0.204853
evm.model.Scaffold2.695	TRB1	0.527±0.178	0.828±0.208	0.000±0.000	0.065675	0.017972
evm.model.Scaffold1.317	TRB2-1	0.696±0.288	0.912±0.122	0.696±0.099	0.162377	0.499611
evm.model.Scaffold274.23	TRB2-2	0.771±0.365	0.761±0.049	0.436±0.202	0.484258	0.127888
evm.model.Scaffold135.18	ARR2	0.296±0.091	0.439±0.226	0.900±0.104	0.196477	0.000867
evm.model.Scaffold154.10	MYB2R-1	0.860±0.122	0.682±0.200	0.384±0.100	0.136028	0.003534
evm.model.Scaffold18.232	PHL7	0.719±0.130	0.895±0.094	0.706±0.271	0.068506	0.471713
evm.model.Scaffold18.233	PHR1-3	0.551±0.508	0.391±0.461	0.692±0.326	0.353850	0.354414
evm.model.Scaffold19.251	GAMYB	0.301±0.151	0.709±0.163	0.589±0.474	0.016797	0.202983
evm.model.Scaffold22.175	MYB15	0.427±0.014	0.661±0.296	0.000±0.000	0.151788	0.000190
evm.model.Scaffold3.158	KAN1	0.679±0.426	0.226±0.288	0.066±0.114	0.105610	0.060944
evm.model.Scaffold32.127	MYB1R1-1	0.660±0.061	0.606±0.138	0.905±0.150	0.289138	0.045460
evm.model.Scaffold3.515	MYB1R1-2	0.590±0.247	0.482±0.449	0.775±0.055	0.368950	0.162075
evm.model.Scaffold32.48	APRR2	0.330±0.128	0.129±0.142	0.522±0.501	0.072030	0.289705
evm.model.Scaffold38.14	ALY2	0.883±0.105	0.581±0.128	0.515±0.094	0.018136	0.005420
evm.model.Scaffold392.31	ALY3	0.755±0.068	0.665±0.214	0.793±0.198	0.276120	0.388189
evm.model.Scaffold43.169	MYBS3	0.532±0.013	0.791±0.200	0.383±0.334	0.076918	0.260641
evm.model.Scaffold44.189	MYB3R-5	0.434±0.227	0.614±0.537	0.586±0.120	0.317472	0.189688
evm.model.Scaffold446.23	SRM1	0.434±0.191	0.851±0.160	0.656±0.250	0.022678	0.146085
evm.model.Scaffold45.28	MYB1R1	0.900±0.095	0.784±0.132	0.727±0.016	0.145111	0.041511

evm.model.Scaffold455.4	PHR1	0.134±0.022	0.545±0.272	0.675±0.282	0.059298	0.039479
evm.model.Scaffold47.46	MYB3R-5	0.551±0.035	0.453±0.008	0.704±0.265	0.016870	0.211158
evm.model.Scaffold5.301	RR21	0.778±0.195	0.637±0.235	0.732±0.149	0.234167	0.380617
evm.model.Scaffold62.114	HHO6	0.583±0.036	0.781±0.190	0.138±0.120	0.104856	0.008353
**NAC**						
evm.model.Scaffold140.42	NACA2	0.937±0.067	0.445±0.095	0.365±0.047	0.001375	0.000253
evm.model.Scaffold15.182	NAC8	0.951±0.044	0.799±0.113	0.549±0.173	0.066412	0.024568
evm.model.Scaffold1.693	NAC2	0.193±0.112	0.473±0.457	0.643±0.221	0.200592	0.026017
evm.model.Scaffold175.42	NAC62	0.699±0.261	0.550±0.160	0.335±0.089	0.227008	0.062611
evm.model.Scaffold19.309	NAC17	0.448±0.107	0.174±0.058	0.801±0.183	0.014292	0.029045
evm.model.Scaffold20.107	NAC89	0.257±0.031	0.607±0.341	0.301±0.195	0.107935	0.367228
evm.model.Scaffold20.54	NACB	0.842±0.171	0.697±0.084	0.503±0.127	0.141092	0.027774
evm.model.Scaffold229.28	NAC72	0.973±0.042	0.000±0.000	0.000±0.000	0.000314	0.000314
evm.model.Scaffold26.134	NAC47	0.160±0.040	0.301±0.088	0.956±0.044	0.046381	0.000011
evm.model.Scaffold289.41	NAC101	0.504±0.144	0.875±0.184	0.592±0.214	0.027234	0.294830
evm.model.Scaffold3.628	NAC78	0.828±0.167	0.644±0.075	0.823±0.182	0.093998	0.486856
evm.model.Scaffold38.154	NAC73	0.818±0.164	0.524±0.124	0.729±0.054	0.036575	0.283654
evm.model.Scaffold51.52	NACB	0.231±0.057	0.622±0.358	0.241±0.136	0.098453	0.420428
evm.model.Scaffold54.46	NAC45	0.656±0.434	0.258±0.230	0.324±0.055	0.127243	0.157818
evm.model.Scaffold56.33	NAC41	0.950±0.067	0.619±0.154	0.358±0.196	0.024016	0.000180
evm.model.Scaffold68.4	NAC56	0.838±0.150	0.629±0.256	0.774±0.196	0.151396	0.338999
evm.model.Scaffold25.232	NAC5	0.169±0.167	0.178±0.307	0.580±0.519	0.484181	0.151058
evm.model.Scaffold257.21	NAC86	0.000±0.000	0.333±0.577	0.140±0.083	0.211325	0.050506
evm.model.Scaffold67.3	NAC91	0.787±0.286	0.134±0.232	0.575±0.499	0.016835	0.280831
**DOF**						
evm.model.Scaffold13.356	DOF4.6	0.863±0.176	0.725±0.169	0.063±0.110	0.190254	0.002383

evm.model.Scaffold39.140	DOF1.8	0.887±0.120	0.819±0.058	0.890±0.100	0.221899	0.486259
evm.model.Scaffold69.14	CDF3	0.587±0.040	0.157±0.273	0.930±0.112	0.054480	0.011776
**HD**						
evm.model.Scaffold3.236	BELL1	0.120±0.207	0.000±0.000	0.540±0.398	0.211325	0.101206
evm.model.Scaffold10.16	MIF2	0.817±0.193	0.513±0.379	0.337±0.155	0.152048	0.015149
evm.model.Scaffold34.72	MIF3	0.798±0.063	0.787±0.191	0.127±0.123	0.463886	0.001842
evm.model.Scaffold27.221	HAT3.1	0.551±0.070	0.862±0.119	0.363±0.059	0.013095	0.012524
evm.model.Scaffold32.119	HAT4	0.940±0.013	0.826±0.198	0.092±0.092	0.211733	0.001685
evm.model.Scaffold114.7	HAT22	0.702±0.459	0.312±0.377	0.306±0.081	0.160418	0.135885
evm.model.Scaffold151.57	HAT22	0.773±0.309	0.299±0.266	0.063±0.110	0.057752	0.022495
evm.model.Scaffold5.249	HOX3	0.921±0.098	0.000±0.000	0.095±0.165	0.001866	0.001870
evm.model.Scaffold21.220	KNAT3	0.825±0.248	0.665±0.035	0.492±0.252	0.189808	0.088681
evm.model.Scaffold26.278	BLH1	0.820±0.165	0.753±0.063	0.692±0.113	0.282518	0.168056
evm.model.Scaffold51.18	BLH1	0.579±0.024	0.587±0.066	0.761±0.217	0.427403	0.140837
evm.model.Scaffold6.225	BLH6	0.842±0.148	0.628±0.073	0.541±0.099	0.056108	0.025230
evm.model.Scaffold201.19	BLH9	0.611±0.337	0.519±0.473	0.307±0.308	0.398864	0.156641
evm.model.Scaffold26.207	WOX11	0.295±0.288	0.178±0.224	0.844±0.254	0.304557	0.034592
evm.model.Scaffold30.134	WOX13	0.544±0.119	0.701±0.061	0.659±0.313	0.067877	0.300470
evm.model.Scaffold320.12	PRH	0.951±0.049	0.768±0.071	0.793±0.136	0.012932	0.086734
evm.model.Scaffold38.162	RLT1	0.906±0.124	0.767±0.076	0.532±0.041	0.093512	0.012727
evm.model.Scaffold54.5	RLT1.1	0.810±0.201	0.816±0.069	0.718±0.156	0.483480	0.282527
evm.model.Scaffold55.37	ATHB8	0.714±0.196	0.743±0.432	0.047±0.082	0.461968	0.008133
evm.model.Scaffold10.15	ZHD9	0.066±0.114	0.611±0.356	0.373±0.359	0.053371	0.136365
**TCP**						
evm.model.Scaffold262.2	TCP2	0.887±0,159	0.485±0.130	0.403±0.056	0.014714	0.012052
evm.model.Scaffold28.221	TCP8	0.344±0.096	0.696±0.264	0.393±0.057	0.067627	0.251836

evm.model.Scaffold7.19	TCP9	0.871±0.022	0.879±0.120	0.604±0.046	0.458428	0.001665
evm.model.Scaffold155.81	TCP13	0.203±0.016	0.454±0.473	0.361±0.180	0.226947	0.133254
evm.model.Scaffold10.275	TCP20	0.739±0.105	0.456±0.476	0.000±0.000	0.205909	0.003306
evm.model.Scaffold18.264	TCP23	0.606±0.203	0.230±0.398	0.963±0.041	0.120608	0.043951
**SBP**						
evm.model.Scaffold9.72	SPL3	0.668±0.045	0.909±0.079	0.397±0.111	0.008472	0.018280
evm.model.Scaffold160.9	SPL7	0.889±0.064	0.738±0.308	0.331±0.057	0.243524	0.000185
evm.model.Scaffold281.35	SPL7.1	0.256±0.021	0.761±0.318	0.117±0.114	0.054822	0.082446
**GRAS**						
evm.model.Scaffold44.37	SCL1	0.456±0.120	0.441±0.314	0.815±0.167	0.473148	0.022097
evm.model.Scaffold20.292	SCL14	0.296±0.216	0.756±0.230	0.537±0.169	0.032793	0.104146
evm.model.Scaffold52.21	SCL33	0.944±0.046	0.978±0.023	0.690±0.040	0.168378	0.001014
evm.model.Scaffold85.71	SCL30	0.163±0.145	0.465±0.504	0.000±0.000	0.205293	0.094666
evm.model.Scaffold6.429	MRS2-3	0.773±0.226	0.639±0.190	0.797±0.326	0.239463	0.461212

Table S9. The binding sites of TFs in the promoters of genes involved in sesquipterpene biosynthesis in *A. sinensis*.

TF	Gene	No.	Site Name	Position	Strand	sequence
bHLH	AACT1	1	G-box	1796	-	CACGTG
		2	MYC	447	-	CATTTG
		3	MYC	1494	-	CATTTG
	AACT2	1	G-box	620	-	CACGAC
		2	G-box	1873	-	CACGAC
		3	MYC	657	-	CATTTG
		4	MYC	1910	-	CATTTG
		5	MYC	878	-	CAATTG
		6	MYC	2132	-	CAATTG
	HMGS	1	MYC	150	+	CATTTG
		2	MYC	2340	-	CATGTG
		3	MYC	1784	-	CATTTG
		4	MYC	2357	+	CATTTG
		5	MYC	1242	-	CATTTG
		6	MYC	2258	+	CATGTG
	HMGR	1	G-box	2308	-	CACGTG
		2	MYC	206	+	CAATTG
		3	MYC	224	+	CAATTG
	MVK	1	MYC	368	+	CAATTG
		2	MYC	1769	-	TCTCTTA
	PMK	1	MYC	1489	+	CATGTG
		2	MYC	1110	+	TCTCTTA
	MDC	1	MYC	1548	-	CAATTG
		2	MYC	1751	-	CATGTG
		3	MYC	1663	+	CATTTG
	DXS1	1	MYC	1702	+	CATTTG
		2	MYC	1475	-	CATTTG
		3	MYC	502	-	CATTTG
	DXS3	1	MYC	899	+	CATTTG
		2	MYC	924	-	CATTTG
		3	MYC	911	+	CATGTG
		4	MYC	1279	-	TCTCTTA
	DXR	1	MYC	77	-	CATTTG
		2	MYC	2070	-	CATGTG
		3	MYC	2056	-	CATTTG
		4	MYC	2389	-	CATGTG
	MCT	1	MYC	264	+	CAATTG
		2	MYC	1135	+	CAATTG

	3	MYC	302	+	CAATTG
	4	MYC	2312	-	CATTTG
MCS	1	MYC	983	-	CATGTG
	2	MYC	66	+	CATGTG
	3	MYC	844	+	CATTTG
	4	MYC	2134	-	CATGTG
	5	MYC	524	-	CATGTG
	6	MYC	1369	-	CAATTG
	7	MYC	910	+	CATTTG
	8	MYC	1127	-	CATGTG
	9	MYC	457	+	CATTTG
	10	MYC	1433	-	CATGTG
	11	MYC	616	+	CAATTG
	12	MYC	1329	+	TCTCTTA
	13	MYC	2206	+	TCTCTTA
	14	MYC	1786	-	TCTCTTA
HDS	1	MYC	817	+	CATGTG
	2	MYC	2030	-	CATTTG
HDR1	1	MYC	872	+	CATTTG
HDR2	1	MYC	1934	-	CAATTG
	2	MYC	413	+	CATTTG
	3	MYC	1486	-	CATTTG
	4	MYC	2328	-	CATTTG
	5	MYC	1703	+	CATTTG
	6	MYC	1854	-	CAATTG
	7	MYC	1383	+	TCTCTTA
IPPI	1	MYC	779	+	CAATTG
	2	MYC	2462	+	CATTTG
	3	MYC	1924	-	CATTTG
	4	MYC	1857	-	CATTTG
	5	MYC	2095	+	CATTTG
	6	MYC	7	+	TCTCTTA
FPS	1	MYC	581	+	CAATTG
	2	MYC	1816	-	CAATTG
	3	MYC	1115	-	CATTTG
	4	MYC	2152	-	CAATTG
	5	MYC	400	-	CATTTG
	6	MYC	1631	-	CATTTG
TPS1	1	MYC	1101	+	TCTCTTA
	2	MYC	1923	+	TCTCTTA
	3	MYC	1307	+	TCTCTTA
	4	MYC	1519	+	TCTCTTA

		5	MYC	204	-	TCTCTTA
		6	MYC	1719	+	TCTCTTA
		7	MYC	646	+	TCTCTTA
		8	MYC	446	+	TCTCTTA
		9	MYC	890	+	TCTCTTA
	TPS2	1	MYC	579	-	CATTTG
		2	MYC	1948	-	CATTTG
		3	MYC	1254	+	CATTTG
		4	MYC	2032	-	CATTTG
		5	MYC	950	+	CAATTG
		6	MYC	1792	-	CATTTG
	TPS3	1	MYC	1262	+	CATTTG
		2	MYC	2117	+	CATGTG
		3	MYC	1822	+	CATGTG
		4	MYC	1550	-	CATTTG
		5	MYC	1905	-	CATTTG
	TPS4	1	MYC	164	-	CATTTG
		2	MYC	2293	+	CATTTG
		3	MYC	1942	-	CAATTG
		4	MYC	2397	-	CATGTG
		5	MYC	344	+	CAATTG
		6	MYC	2117	-	CAATTG
bZIP	AACT1	1	ABRE	119	-	AACCCGG
		2	ABRE	1796	-	CACGTG
		3	G-box	1620	-	CACGTG
	AACT2	1	G-box	620	-	CACGAC
		2	G-box	1873	-	CACGAC
	HMGS	1	G-box	2255	-	TCCACATGGCA
	HMGR	1	C/G-box	2344	-	GGTGACGTGGCA
		2	C/G-box	460	-	TgTGACGTGGtA
		3	G-box	462	+	CACGTC
		4	G-box	2346	+	CACGTC
		5	G-box	2308	-	CACGTG
		6	ABRE	2309	+	ACGTG
	MVK	1	ABRE	1354	+	AACCCGG
		2	ABRE	2222	+	CCCcCGTGGC
		3	ABRE	2299	-	GACACGTGGC
		4	ABRE	2300	+	GCCACGTGaC
		5	G-box	99	+	CACGAC
	PMK	1	hor1-box	2159	+	GTGAGTCAT
	MDC	1	ABRE	205	+	TACGTG
		2	ABRE	205	-	CACGTA

	DXS1	1	ABRE	2363	+	ACGTG
		2	ABRE	107	+	TACGTG
		3	G-Box	2362	-	CACGTT
		4	G-box	2138	-	GCCACGTGGA
		5	G-box	106	-	TAACACGTAG
	DXS3	1	ABRE	1620	-	CACGTG
		2	ABRE	1621	+	ACGTG
	DXR	1	ABRE	1735	-	TACGTG
		2	ABRE	1735	+	CACGTA
	MCT	1	G-box	75	-	CACGTC
		2	ABRE	76	+	ACGTG
		3	ABRE	2305	-	CACGTG
		4	ABRE	1685	-	GACACGTGGC
		5	ABRE	2306	+	ACGTG
	CMK	1	ABRE	2262	+	ACGTG
		2	ABRE	2318	-	ACGTG
		3	G-Box	2261	-	CACGTT
		4	G-Box	2318	+	CACGTT
	MCS	1	ABRE	2386	+	ACGTG
		2	G-Box	63	-	TCCACATGGCA
		3	G-Box	2385	-	CACGTT
	HDS	1	ABRE	2160	-	ACGTG
		2	G-Box	2160	+	CACGTT
	HDR1	1	ABRE	2399	+	ACGTG
		2	G-Box	2398	-	CACGTT
	HDR2	1	ABRE	2482	-	GACACGTACGT
		2	ABRE	2485	+	TACGTG
		3	ABRE	2485	-	CACGTA
	TPS2	1	G-Box	1328	-	TCACGGTtCAC
	TPS3	1	ABRE	232	+	ACGTG
		2	ABRE	2298	-	ACGTG
		3	G-Box	231	-	CACGTT
		4	G-box	2298	+	CACGTC
	TPS4	1	ABRE	620	-	ACGTG
		2	G-Box	620	+	CACGTT
		3	G-Box	2395	+	TCCACATGGCA
		4	G-box	970	-	CACGAC
		5	G-box	2447	+	CACGAC
WRKY	AACT1	1	W-box	30	-	TTGACC
		2	W-box	507	+	TTGACC
		3	W-box	313	-	TTGACC
	HMGR	1	W-box	227	+	TTGACC

	MVK	1	W-box	1101	+	TTGACC
		2	W-box	2240	-	TTGACC
		3	W-box	1032	+	TTGACC
		4	W-box	2075	+	caGTTGACTATCA
	PMK	1	W-box	1240	-	TTGACC
		2	W-box	2131	-	TTGACC
		3	W-box	2121	+	TTGACC
		4	W-box	2305	+	TTGACC
	DXS1	1	W-box	74	-	GGTTGACTGACc
		2	W-box	88	+	TTGACC
		3	W-box	2313	-	GaTTTGACTATTG
	DXS3	1	W-box	1518	+	TTGACC
	DXR	1	W-box	742	+	TTTCTGACTATT
		2	W-box	1140	+	TTTCTGACTATT
	MCT	1	W-box	1041	-	TTGACC
		2	W-box	1501	+	TTGACC
	MCS	1	W-box	1262	+	TTGACC
	HDR2	1	W-box	1960	-	TTGACC
	IPPI	1	W-box	1355	+	TTGACC
		2	W-box	1812	+	TTGACC
	TPS1	1	W-box	2088	-	TTGACC
	TPS3	1	W-box	486	+	TTGACC
	TPS4	1	W-box	1844	-	TTGACC
ERF	AACT1	1	DRE	2377	-	ACCGAGA
	AACT2	1	DRE	948	+	GCCGAC
	HMGR	1	GCC-box	767	-	AGCCGCC
	MVK	1	DRE	1145	-	GCCGAC
	PMK	1	GCC-box	2407	+	AGCCGCC
		2	DRE	2105	-	CAAttcTGTCGGTTcATTTT
	DXR	1	DRE	2211	+	GCCGAC
	CMK	1	GCC-box	1607	+	GCAGCCcCT
	HDR1	1	DRE	2478	-	GCCGAC
	TPS1	1	GCC-box	2240	+	AGCCGCC
MYB	AACT1	1	MBSI	923	+	TTTTTACGGTTA
		2	MYB	1024	+	TAACCA
		3	MYB	2127	-	CAACCA
		4	MYB	1374	+	CAACCA
		5	MYB	1830	+	CCGTTG
		6	MYB	929	-	TAACTG
		7	MYB	1500	+	TAACTG
		8	MYB	1215	-	TAACTG
	AACT2	1	MBS	450	-	CAACTG

	2	MBS	1703	-	CAACTG
	3	MRE	602	+	AACCTAA
	4	MRE	1855	+	AACCTAA
	5	MYB	521	-	CAACAG
	6	MYB	1925	-	CAACAG
	7	MYB	711	-	CAACCA
	8	MYB	1964	-	CAACCA
	9	MYB	672	-	CAACAG
	10	MYB	1774	-	CAACAG
	11	MYB	2457	-	TAACTG
	12	MYB	1855	-	gAAAGTTAGGTTA
	13	MYB	602	-	gAAAGTTAGGTTA
HMGS	1	MBS	66	+	CAACTG
	2	MRE	934	-	AACCTAA
	3	MRE	1477	-	AACCTAA
	4	MYB	697	-	CAACCA
	5	MYB	1691	+	CAACAG
	6	MYB	1150	+	CAACAG
	7	MYB	2336	+	CAACCA
	8	MYB	1777	+	TAACTG
	9	MYB	1235	+	TAACTG
	10	MYB	2147	-	TAACTG
HMGR	1	MYB	810	+	CAACCA
	2	MYB	2429	+	CAACCA
	3	MYB	1110	+	CAACAG
	4	MYB	1113	-	TAACTG
	5	MYB	1505	+	TAACTG
	6	MYB	2307	+	CcCACGTGCT
MVK	1	MBS	1086	+	CAACTG
	2	MBS	1760	+	CAACTG
	3	MBS	1385	-	CAACTG
	4	MBS	2074	-	CAACTG
	5	MYB	454	+	CAACCA
	6	MYB	1192	-	CAACCA
	7	MYB	1058	+	CAACCA
	8	MYB	467	+	CAACCA
	9	MYB	1177	-	CAACCA
	10	MYB	335	-	TAACTG
PMK	1	MBS	2126	-	CAACTG
	2	MBSI	821	+	aaaAaaC(G/C)GTTA
	3	MYB	680	+	CAACCA
	4	MYB	2061	-	TAACCA

	5	MYB	1099	-	CAACCA
	6	MYB	2134	+	CAACCA
	7	MYB	2061	-	TAACCA
	8	MYB	1987	+	TAACTG
	9	MYB	2126	-	CAACTG
MDC	1	MRE	1966	+	AACCTAA
	2	MYB	2419	+	TAACCA
	3	MYB	2194	-	CCGTTG
	4	MYB	2350	-	cGTGGTcGGTGG
	5	MYB	2457	+	TCTCtCCTACC
DXS1	1	MRE	692	+	AACCTAA
	2	MRE	1724	-	AACCTAA
	3	MYB	1843	-	TAACCA
	4	MYB	1103	-	TAACCA
	5	MYB	626	+	CAACCA
	6	MYB	211	+	TAACCA
	7	MYB	3	-	CAACCA
	8	MYB	312	-	CCGTTG
DXS3	1	MRE	109	+	AACCTAA
	2	MYB	148	+	CAACCA
	3	MYB	1042	+	CAACAG
	4	MYB	403	+	CAACCA
	5	MYB	1863	-	TAACCA
	6	MYB	527	+	CCGTTG
	7	MYB	2059	+	CCGTTG
	8	MYB	1863	-	TAACCA
	9	MYB	2032	-	TAACTG
	10	MYB	2046	-	TAACTG
	11	MYB	2039	-	TAACTG
	12	MYB	2192	+	CCGTTG
	13	MYB	2223	-	TAACTG
DXR	1	MYB	798	-	TAACCA
	2	MYB	1628	-	CAACCA
	3	MYB	1627	+	GGTGGTtGGTGG
	4	MYB	812	+	TAACCA
	5	MYB	525	-	TAACTG
	6	MYB	866	-	TAACTG
	7	MYB	602	+	TAACTG
CMK	1	MRE	2001	-	AACCTAA
	2	MYB	2101	+	CAACCA
	3	MYB	2116	+	TAACTG
MCS	1	MYB	27	-	CAACAG

	2	MYB	1936	-	CAACAG
	3	MYB	2264	+	TAACCA
	4	MYB	1037	-	TAACCA
	5	MYB	397	+	TAACCA
	6	MYB	1983	-	TAACCA
	7	MYB	1214	-	TAACCA
	8	MYB	350	-	CAACCA
	9	MYB	582	-	CAACCA
	10	MYB	261	+	TAACTG
	11	MYB	1712	-	CAACTG
	12	MYB	835	+	TAACTG
	13	MYB	1775	-	CAACTG
	14	MYB	446	-	CAACTG
	15	MYB	1066	-	TAACTG
	16	MYB	284	-	GGTGGTgGGTGt
HDS	1	MRE	1371	-	AACCTAA
	2	MYB	855	+	TAACCA
	3	MYB	1953	-	TAACCA
	4	MYB	1633	+	TAACCA
	5	MYB	2001	+	CCGTTG
	6	MYB	855	+	TAACCA
	7	MYB	1953	-	TAACCA
	8	MYB	1633	+	TAACCA
	9	MYB	2432	+	TAACTG
	10	MYB	2463	+	TAACTG
HDR1	1	MBSI	1036	+	aaaAaaC(G/C)GTTA
	2	MRE	1844	-	AACCTAA
	3	MYB	495	-	CAACCA
	4	MYB	946	+	TAACCA
	5	MYB	1472	+	TAACCA
	6	MYB	1448	+	CAACCA
HDR2	1	MRE	505	+	AACCTAA
	2	MYB	1593	+	CAACCA
	3	MYB	1774	-	TAACCA
	4	MYB	112	+	CAACCA
	5	MYB	918	+	CAACCA
	6	MYB	1248	+	CAACCA
	7	MYB	1576	+	CAACAG
	8	MYB	1774	-	TAACCA
IPPI	1	MBS	1032	+	CAACTG
	2	MBS	2213	+	CAACTG
	3	MYB	799	-	CAACCA

	4	MYB	1689	+	CAACCA
	5	MYB	1373	+	TAACCA
	6	MYB	1730	-	CAACCA
	7	MYB	801	-	ACCACAACC
FPS	1	MBS	2447	+	CAACTG
	2	MRE	853	-	AACCTAA
	3	MYB	375	-	TAACCA
	4	MYB	2458	+	TAACCA
	5	MYB	987	+	TAACCA
	6	MYB	1812	+	TAACCA
	7	MYB	577	+	TAACCA
	8	MYB	1915	-	CAACCA
	9	MYB	1606	-	TAACCA
	10	MYB	450	+	CAACAG
	11	MYB	682	-	CAACCA
	12	MYB	738	-	CCGTTG
TPS1	1	MYB	13	-	TAACCA
	2	MYB	1328	-	TAACCA
	3	MYB	912	-	TAACCA
	4	MYB	1740	-	TAACCA
	5	MYB	467	-	TAACCA
	6	MYB	1540	-	TAACCA
	7	MYB	1122	-	TAACCA
	8	MYB	2091	+	CAACAG
	9	MYB	267	-	TAACCA
	10	MYB	667	-	TAACCA
TPS2	1	MBS	2081	-	CAACTG
	2	MRE	957	+	AACCTAA
	3	MRE	2450	+	AACCTAA
	4	MYB	267	+	CAACCA
	5	MYB	2479	+	CAACAG
	6	MYB	1387	+	CAACCA
	7	MYB	1548	+	CAACAG
	8	MYB	1095	+	TAACCA
	9	MYB	1915	+	CAACAG
	10	MYB	1502	+	CAACAG
	11	MYB	1690	+	TAACCA
	12	MYB	1031	+	CAACCA
	13	MYB	2259	+	TAACCA
	14	MYB	1379	+	CAACCA
	15	MYB	2184	+	TAACTG
TPS3	1	MYB	388	+	TAACCA

		2	MYB	1519	-	CAACCA
		3	MYB	777	-	CAACAG
		4	MYB	2399	+	CAACCA
		5	MYB	742	-	TAACCA
		6	MYB	1191	-	CAACCA
		7	MYB	34	-	CCGTTG
		8	MYB	247	+	CCGTTG
		9	MYB	388	+	TAACCA
		10	MYB	742	-	TAACCA
		11	MYB	2437	+	TAACTG
		12	MYB	777	-	CAACAG
		13	MYB	1220	+	GaAGGTtGGTGG
	TPS4	1	MBS	1205	-	CAACTG
		2	MBS	1601	-	CAACTG
		3	MRE	1620	-	AACCTAA
		4	MRE	2411	+	AACCTAA
		5	MRE	1703	-	AACCTAA
		6	MYB	1438	+	CAACCA
		7	MYB	1909	+	TAACCA
		8	MYB	1826	+	CAACCA
		9	MYB	1909	+	TAACCA
		10	MYB	1159	+	TAACTG
		11	MYB	2352	-	TAACTG
		12	MYB	1241	+	TAACTG
		13	MYB	1205	-	CAACTG
		14	MYB	1601	-	CAACTG
NAC	HMGS	1	NAC	746	-	CCtAACCTTCTT
	CMK	1	NAC	2032	-	tCTGCCGGcGA
	TPS4	1	NAC	985	+	GCTGCCGGAGA
DOF	AACT2	1	DOF	2486	-	AAAAAAGAGA
	DXS3	1	DOF	1991	+	AtaAAAGAAAGAAA
		2	DOF	1036	+	AGCAAAGCAA
		3	DOF	568	+	CTAAAAAGAAT
	MCT	1	DOF	729	+	gAGAAAGAAAGAgA
HD	AACT1	1	HD	428	-	AGCTaCTCCTA
		2	HD	1648	+	TGACTGATTGAT
	HMGS	1	HD-Zip	614	+	CAAT(A/T)ATTG
	MVK	1	HD	943	+	AGGATCGATGg
		2	HD-Zip	1620	-	CAAT(A/T)ATTG
	CMK	1	HD	2345	-	CCTCTCttGTC
	HDR1	1	HD-Zip	428	+	CAAT(A/T)ATTG
TCP	MDC	1	TCP	2346	-	GTGGGCCC

MADS	DXR	1	MADS-box	1507	-	CTTTTTTTGG
	IPPI	1	MADS-box	132	+	CTTTTTTTGG

Supplementary methods

Detecting the volatile oil of *P. rubrigenum* hyphae and fermentation media. To detect the volatile compounds of *P. rubrigenum*, fresh cultures of *P. rubrigenum* were inoculated into a malt extract broth media and cultured at 25 °C on a rotary shaker at a speed of 120 rpm for 5 and 10 days. Three replicates using various liquid media were used for each group.

The volatile oil of *P. rubrigenum* hyphae was extracted as follows. *P. rubrigenum* hyphae (1.0 g) were weighed and placed in a 50 mL centrifuge tube, and 10.0 mL ethyl acetate was added to the tube. *P. rubrigenum* hyphae were incubated overnight at room temperature and extracted for 45 min using the 40 kHz ultrasonic cold extraction method. The upper solvent phase was separated by centrifugation at 7000 rpm for 10 min at 4 °C. After adding ethyl acetate to supplement weightlessness, the volatile oil was filtered by 0.22 μm PTFE filter membrane and then stored in a dark glass bottle at 4 °C prior to GC-MS analysis. The GC-MS method was similar to that described above.

Volatile oil from the fermentation media of *P. rubrigenum* was extracted as follows. *P. rubrigenum* fermentation media was filtered through gauze to remove hyphae, and 150 mL of the fermentation media was transferred to a new Erlenmeyer flask. Thereafter, 150 mL of ethyl acetate was added. The solvent was entirely volatile after precise addition to 500 uL of ethyl acetate and mixed properly by vortexing after overnight incubation under nitrogen to concentrate the sample. Prior to GC-MS analysis, the volatile oil from the fermentation media was filtered through a 0.22 μm PTFE filter membrane and stored in a dark glass bottle at 4 °C. The GC-MS method was similar to

that described above.

Quantitative proteomics by Tandem Mass Tags (TMT) method. Samples were ground in liquid nitrogen and lysed with SDT lysis buffer (4% SDS, 100 mM DTT, and 10 mM TEAB), followed by 5 min of ultrasonication on ice. After incubation at 95 ºC for 8 min, the lysate was centrifuged at 12000 × *g* for 15 min at 4 ºC. The supernatant was reduced with 10 mM DTT for 1 h at 56 ºC and subsequently alkylated with iodoacetamide for 1 h at room temperature in the dark. Thereafter, four times the volume of precooled acetone was added, the samples mixed by vortexing, and incubated at -20 ºC for at least 2 h. Samples were then centrifuged at 12000 × *g* for 15 min at 4 ºC, and the pellet was collected. After washing with 1mL cold acetone, the pellet was dissolved in a dissolution buffer (8 M urea, 100 mM TEAB, pH 8.5).

The volume of each protein sample was adjusted to 100 µL with DB dissolution buffer (8 M urea, 100 mM TEAB, pH 8.5). Trypsin and 100 mM TEAB buffer were added, and the sample was mixed and digested at 37 °C for 4 h. Trypsin and $CaCl_2$ were then added, and the sample was digested overnight. Formic acid was used to adjust the pH to below 3, and centrifuged at 12,000 × *g* for 5 min at room temperature. The supernatant was loaded onto a C18 desalting column, washed with three column volumes of wash buffer (0.1% formic acid, 3% acetonitrile), and then eluted with elution buffer (0.1% formic acid and 70% acetonitrile). The eluents from each sample were collected and lyophilized, reconstituted in 100 µL of 0.1 M TEAB buffer, and 41 µL of acetonitrile-dissolved TMT labeling reagent was added. Sample were mixed by

shaking for 2 h at room temperature. The reaction was stopped by adding 8% ammonia. All labeling samples were mixed with equal volume, desalted, and lyophilized.

For transition library construction, shotgun proteomics analyses were performed using an EASY-nLC^{TM} 1200 UHPLC system (Thermo Fisher) coupled with a Q Exactive^{TM} HF-X mass spectrometer (Thermo Fisher) operating in the data-dependent acquisition (DDA) mode. A 1 μg sample was injected onto a homemade C18 Nano-Trap column (4.5 cm × 75 μm, 3 μm). Peptides were separated on a homemade analytical column (15 cm × 150 μm, 1.9 μm) using linear gradient elution: 0 min, 94% mobile phase A (2% acetonitrile, adjusted pH to 10.0, using ammonium hydroxide), 6% mobile phase B (98% acetonitrile); 2 min, 85% mobile phase A, 15% mobile phase B; 78.5 min, 60% mobile phase A, 40% mobile phase B. The separated peptides were analyzed by Q Exactive^{TM} HF-X mass spectrometer (Thermo Fisher), with ion source of Nanospray Flex^{TM} (ESI), spray voltage of 2.3 kV and ion transport capillary temperature of 320 °C. The full scan ranged from m/z 350 to 1500 with a resolution of 60000 (at m/z 200), an automatic gain control (AGC) target value was 3×10^6, and the maximum ion injection time was 20 ms. The 40 precursors with the highest abundance in the full scan were selected and fragmented by higher-energy collisional dissociation (HCD) and analyzed by MS/MS, with a resolution of 45000 (at m/z 200) for 10 plex, the automatic gain control (AGC) target value was 5×10^4 the maximum ion injection time was 86 ms, the normalized collision energy was set as 32%, the intensity threshold was 1.2×10^5, and the dynamic exclusion parameter was 20 s.

The resulting spectra from each run were searched separately against the peptide database translated from *A. sinensis* genome (NCBI SRA: PRJNA556948) by the search engines: Proteome Discoverer 2.4 (PD 2.4, Thermo). The searched parameters were set as follows: mass tolerance for the precursor ion was 10 ppm, and mass tolerance for the product ion was 0.02 Da. Carbamidomethyl was used as fixed modifications. Oxidation of the methionine (M) and TMT plexes was specified as a dynamic modification. Acetylation, TMT plex, Met-loss, and Met-loss+ acetyl were specified as N-Terminal modifications in PD 2.4. A maximum of two missed cleavage sites were allowed.

To improve the quality of the analysis results, the PD 2.4 software further filtered the retrieval results, and peptide spectrum matches (PSMs) with a credibility of more than 99% were identified as PSMs. The identified proteins contained at least one unique peptide. The identified PSMs and proteins were retained and analyzed with an FDR of no more than 1.0%. Protein quantitation results were statistically analyzed using a t-test.

Gene Ontology (GO) and InterPro (IPR) functional analysis were conducted using the interproscan program against the non-redundant protein database (including Pfam, PRINTS, ProDom, SMART, ProSite, PANTHER). The databases of COG (Clusters of Orthologous Groups) and KEGG (Kyoto Encyclopedia of Genes and Genomes) were used to analyze the protein family and pathway. The DEPs were used for volcanic map analysis, cluster heat map analysis, and enrichment analysis of GO, IPR and KEGG.

Quantitative phosphoproteome. The samples were individually minced with liquid nitrogen and lysed in lysis buffer containing 100 mM NH_4HCO_3 (pH 8), 6 M urea, and 0.2% SDS, followed by 5 min of ultrasonication on ice. The lysate was centrifuged at 12,000 × *g* for 15 min at 4 °C, and the supernatant was transferred to a clean tube. The protein concentration was determined using the Bradford protein assay. Extracts from each sample were reduced with 2 mM DTT for 1 h at 56 °C and subsequently alkylated with iodoacetamide for 1 h at room temperature in the dark. Thereafter, four times the volume of precooled acetone was mixed with the samples by well vortexing and incubated at -20 °C for at least 2 h. Samples were then centrifuged, and the precipitate was collected. After washing twice with cold acetone, the pellet was dissolved in a dissolution buffer， which contained 0.1 M TEAB (pH 8.5) and 6 M urea. The protein concentration was determined using the Bradford protein assay.

The supernatant from each sample containing 5 mg of protein was digested with Trypsin Gold (Promega) at a 1:50 enzyme-to-substrate ratio. After 16 h of digestion at 37 °C, the peptides were desalted using a C18 cartridge to remove the high urea, and the desalted peptides were dried by vacuum centrifugation.

Selected fractions were redissolved in 250 mM acetic acid with 30% acetonitrile, and the pH was adjusted to 2.5-3.0 with 1 M HCl. Enrichment was carried out using a PHOS-select iron affinity gel (Sigma, P9740), following the manufacturer's instructions. The bound peptides were eluted, dried, and desalted using peptide desalting spin columns (Thermo Fisher Scientific, 89852).

Shotgun proteomics analyses were performed using an EASY-nLC^{TM} 1200 UHPLC system (Thermo Fisher) coupled with an Orbitrap Q Exactive HF-X mass spectrometer (Thermo Fisher) operating in data-dependent acquisition (DDA) mode. A sample volume containing 1 μg of total peptides reconstituted in 0.1% FA was injected onto a homemade C18 Nano-Trap column (2 cm × 100 μm, 3 μm). Peptides were separated on a homemade analytical column (15 cm × 150 μm, 1.9 μm) using a 120 min linear gradient from 5 to 100% of eluent B (0.1% FA in 80% ACN) in eluent A (0.1% FA in H_2O) at a flow rate of 600 nL·min^{-1}. The detailed solvent gradient was as follows: 5-10% B for 2 min; 10-40% B for 105 min; 40-50% B for 5 min; 50-90% B for 3 min; 90-100% B for 5 min.

The Q-Exactive HF-X mass spectrometer was operated in positive polarity mode with a spray voltage of 2.3 kV and a capillary temperature of 320 °C. Full MS scans ranging from 350 to 1500 m/z were acquired at a resolution of 60000 (at 200 m/z) with an automatic gain control (AGC) target value of 3×10^6 and a maximum ion injection time of 20 ms. The 40 most abundant precursor ions from the full MS scan were selected for fragmentation using higher-energy collisional dissociation (HCD) fragment analysis at a resolution of 15000 (at 200 m/z) with an AGC target value of $5e^4$, a maximum ion injection time of 80 ms, normalized collision energy of 27%, an intensity threshold of $1.3e^4$, and a dynamic exclusion parameter of 30 s.

The resulting spectra from each fraction were searched separately against the peptide database translated from the *A. sinensis* genome (NCBI SRA: PRJNA556948) by the search engines: Proteome Discoverer 2.2 (PD 2.2, thermo). The searched

parameters as follows: a mass tolerance of 10 ppm for precursor ion scans and a mass tolerance of 0.02 Da for the product ion scans were used. Carbamidomethyl was specified in PD 2.2 as a fixed modifications. Oxidation of methionine (M), phosphorylation of serine (S), threonine (T) and tyrosine (Y), and acetylation of the N-terminus were specified in PD 2.2, as variable modifications. A maximum of two miscleavage sites were allowed. For protein identification, proteins with at least one unique peptide were identified at an FDR less than 1.0% at the peptide and protein levels, respectively. Proteins containing similar peptides and could not be distinguished based on MS/MS analysis were grouped separately as protein groups. Precursor quantification based on the intensity was used for label-free quantification.

Gene Ontology (GO) and InterPro (IPR) analyses were conducted using the interproscan-5 program against the non-redundant protein database (including Pfam, PRINTS, ProDom, SMART, ProSiteProfiles, and PANTHER), and the Clusters of Orthologous Groups (COG) and Kyoto Encyclopedia of Genes and Genomes (KEGG) database were used to analyze the protein family and pathway.
